# Iron deficiency anaemia in mothers and infants with high inflammatory burden: Prevalence and profile in a South African birth cohort

**DOI:** 10.1371/journal.pgph.0004174

**Published:** 2025-07-09

**Authors:** Jessica E. Ringshaw, Michal R. Zieff, Sadeeka Williams, Chloë A. Jacobs, Zayaan Goolam Nabi, Thandeka Mazubane, Marlie Miles, Donna Herr, Daniel C. Alexander, Melissa Gladstone, Vanja Klepac-Ceraj, Laurel J. Gabard-Durnam, Dima Amso, William P. Fifer, Derek K. Jones, Dan J. Stein, Steven C.R. Williams, Kirsten A. Donald, on behalf of the Khula South Africa Study Team

**Affiliations:** 1 Department of Paediatrics and Child Health, Red Cross War Memorial Children’s Hospital, University of Cape Town, Cape Town, South Africa; 2 Neuroscience Institute, University of Cape Town, Cape Town, South Africa; 3 Centre for Neuroimaging Sciences, Department of Neuroimaging, Kings College London, London, England, United Kingdom; 4 Hawkes Institute and Department of Computer Science, University College London, London, England, United Kingdom; 5 Department of Women and Children’s Health, Institute of Life Course and Medical Science, Alder Hey Children’s NHS Foundation Trust, University of Liverpool, Liverpool, England, United Kingdom; 6 Department of Biological Sciences, Wellesley College, Wellesley, Massachusetts, United States of America; 7 Department of Psychology, Northeastern University, Boston, Massachusetts, United States of America; 8 Department of Psychology, Columbia University, New York, New York, United States of America; 9 Department of Psychiatry, Irving Medical Center, Columbia University, New York, New York, United States of America; 10 Division of Developmental Neuroscience, New York State Psychiatric Institute, New York, New York, United States of America; 11 Department of Pediatrics, Irving Medical Center, Columbia University, New York, New York, United States of America; 12 Cardiff University Brain Research Imaging Centre, Cardiff University, Cardiff, Wales, United Kingdom; 13 South African Medical Research Council (SAMRC), Unit on Risk and Resilience in Mental Disorders, Department of Psychiatry, University of Cape Town, Cape Town, South Africa; Yale University, UNITED STATES OF AMERICA

## Abstract

The scarcity of epidemiological data on anaemia in low- and middle-income countries, coupled with contrasting approaches to the assessment of iron status with inflammation, represent critical research gaps. This study characterised the prevalence and profile of iron deficiency anaemia, including adjustment for inflammation, in mothers and infants from South Africa. Mother-child dyads (*n* = 394) were recruited (2021–2022) for the Khula birth cohort in Cape Town. Haematological metrics, iron metrics, and inflammatory biomarkers were obtained from mothers antenatally and 3–6 months postnatally, and infants 3–18 months postnatally. The extent to which inflammation impacted iron deficiency was assessed using two methods; Method A: higher serum ferritin thresholds for classifying iron status in participants with inflammation (World Health Organisation), Method B: Biomarkers Reflecting Inflammation and Nutritional Determinants of Anaemia (BRINDA) regression which corrects serum ferritin based on inflammatory biomarker concentrations. Prevalence of maternal anaemia was 34.74% (107/308) in pregnancy and 22.50% (54/240) in mothers at 3–6 months after childbirth. Of their infants, 46.82% (125/267) and 48.10% (136/283) were anaemic by 6–12 months and 12–18 months, respectively. Using Method A, the prevalence of maternal iron deficiency (regardless of anaemia), increased from 18.35% (20/109) to 55.04% (60/109) in pregnancy, and from 11.97% (28/234) to 46.58% (109/234) postnatally. Similarly, using Method B, maternal iron deficiency prevalence increased to 38.53% (42/109) in pregnancy, and 25.21% (59/234) postnatally. In infants at 12–18 months, the prevalence of iron deficiency increased from 19.79% (19/96) to 31.25% (30/96) and 32.29% (31/96) using Methods A and B, respectively. Approximately half of anaemia cases in mothers antenatally (50%; 20/40) and postnatally (45.10%; 23/51), and infants at 12–18 months (55.56%; 10/18), were attributable to iron deficiency. This is one of the first studies reporting the extent to which iron deficiency anaemia may be underestimated if inflammation is unaccounted for in South African mothers and infants.

## Introduction

Anaemia, a haematological disorder diagnosed by low serum haemoglobin, affects approximately 1.8 billion people worldwide, with women and children being the most susceptible [1–3]. This public health concern is prominent in low- and middle-income countries (LMICs) where the prevalence of anaemia is disproportionately high due to various contributing factors such as poverty, food insecurity, malnutrition, and infectious disease [4–7]. In comparison to high-income countries where 15% of pregnant women and 10% of young children (6–59 months) are estimated to be anaemic, corresponding statistics in Africa and South Asia range between 30–50% and 35–55%, respectively [1]. South Africa is no exception with one third of women of reproductive age (aged 15 – 49 years; Demographic and Health Survey; SADHS) [8] and between 29% and 42.7% of pregnant women found to be anaemic across regions [9,10]. The high burden of anaemia is evident despite routine antenatal iron supplementation and food fortification initiatives [9–13], making it a health condition for ongoing prioritisation in research, policy, and clinical practice.

Concurrent with an increased focus on antenatal maternal anaemia as an important driver of poor developmental outcomes [14], novel neuroimaging research has provided evidence for an association between maternal anaemia in pregnancy and structural child brain alterations [12] that persist with age [13]. This supports a growing need to understand the aetiology of this complex condition, and to provide deeper insight into the profile and burden of anaemia in LMICs. In recognising anaemia as a priority for targeted prevention and intervention, it has been included in policy briefs [15,16] including the World Health Organisation (WHO) Global Nutrition Targets 2025 [16]. However, efforts to reduce the prevalence of anaemia stagnated between 2000 and 2019 [1], with progress being made at half the pace of other nutritional indicators for maternal and child health [17]. Consequently, and in alignment with the United Nations (UN) Sustainable Development Goals (SDGs) [18], the WHO and UN International Emergency Fund (UNICEF) proposed an extension of nutrition targets to 2030 [19] and developed a framework for accelerated anaemia reduction [2].

This WHO framework emphasizes the complex aetiology of anaemia, identifying the main direct causes as micronutrient deficiencies, chronic infection and inflammation, gynaecological and obstetric conditions, and inherited blood disorders [2]. Given that iron deficiency is reported as accounting for more than 50% of anaemia cases, it has been the most common focus in previous work. Iron deficiency anaemia (IDA) occurs when haemoglobin production is limited by a chronic negative iron balance, representing either an absolute or functional iron deficiency [20]. Absolute iron deficiency is characterized by a total reduction in iron stores due to increased demand, decreased intake, malabsorption, or chronic blood loss [21–23]. While decreased intake is typically due to poor nutrition, instances of increased demand include pregnancy, and malabsorption may be attributable to other factors including alcohol use. In contrast, functional iron deficiency refers to adequate iron stores but insufficient bioavailability of iron. This may be observed in disease pathologies associated with inflammation due to increased iron sequestration [21–23]. Therefore, in communities with an overlapping high prevalence of nutritional risk, substance use, and infectious disease such as Human Immunodeficiency Virus (HIV), the profile of IDA may be complex and require more nuanced assessment and intervention strategies.

In order to effectively reduce the prevalence of anaemia, the WHO recommends five action areas with the first being to analyse data on direct causes and underlying risk factors for anaemia [2]. In addition to assessing anaemia using haemoglobin measures, other data including iron metrics and inflammatory biomarkers are important in establishing the context-specific drivers and local risk factors for anaemia. Metrics typically used to assess iron status, such as serum ferritin and soluble transferrin receptor (sTfR), are altered by inflammatory mechanisms associated with the body’s acute-phase response to infection [24–26]. To account for this, approaches include adjusted serum ferritin thresholds for confirming iron deficiency where there is evidence of inflammation (WHO) [27] or the use of regression models to correct continuous concentrations for iron status metrics (serum ferritin and sTfR) based on inflammatory biomarker concentrations [28,29]. The latter has been developed by Biomarkers Reflecting Inflammation and Nutritional Determinants of Anemia (BRINDA) [30], an ongoing international collaboration to inform global anaemia guidelines on micronutrient assessment and anaemia characterisation [31]. While efforts to refine the BRINDA model are ongoing, reproducible work using this correction approach has demonstrated higher estimates of iron deficiency in children and non-pregnant women after accounting for inflammation [28]. It is particularly relevant in countries with a high burden of infection [32], with findings separately demonstrated across Africa in children with Malaria [33,34] and pregnant women [35].

However, there is an ongoing need for further research on a broader range of infectious and pro-inflammatory exposures. This may be particularly important in South Africa where anaemia and iron deficiency have been found to be more common in HIV-positive pregnant women [9,10], with a recent study suggesting the risk of anaemia, iron deficiency, and IDA to be two fold in this group [11]. Given that 23.5% of women of reproductive age in South Africa are estimated to be living with HIV [36], the role of this infection in the aetiology of anaemia requires further understanding. However, global population research estimating the prevalence of iron deficiency in children and women of reproductive age with adjustment for inflammation do not include pregnancy data and South Africa is not represented in pooled population analyses [37]. Therefore, very little is known regarding the typical ranges for iron metrics across trimesters in this context. This is relevant given that most references ranges for anaemia and iron-deficiency are derived from iron-supplemented women in high-income countries, and may be inappropriate for use in communities of African descent with a genetic predisposition for naturally lower haemoglobin concentrations [3].

Given the relative lack of epidemiological data in LMICs [1,37], the poor characterisation of context-specific overlapping risk factors including iron deficiency in high-risk communities [2], and limitations in the assessment of biomarkers for iron status within the context of inflammation [28], precise reporting using comprehensive data on iron deficiency and anaemia is needed. Furthermore, the widespread use of microcytic anaemia classification (based on mean corpuscular volume; MCV) as a proxy for iron deficiency anaemia in clinical practice [38] requires further validation, particularly in settings with a high burden of infection, through comparison with iron metrics that have been adjusted for inflammation. The aim of this birth cohort study is to contribute towards a global effort to characterise the prevalence and profile of IDA in mothers (during and after pregnancy) and infants using haematological metrics, iron metrics, and inflammatory biomarkers from South Africa. Overall, this could potentially impact public health policies around screening and supplementation before and during pregnancy while simultaneously informing the optimisation of multifactorial prevention and intervention strategies for IDA in mothers and children [3,11,39].

## Methods

### Ethics statement

The Khula South Africa birth cohort study received ethical approval from the University of Cape Town Human Research Ethics Committee (HREC; 666/2021; 782/2022). All mothers provided written informed consent for cohort participation at Khula enrolment, and for their children to participate in study-specific procedures across timepoints. Given the longitudinal nature of the cohort, study consent was obtained from mothers annually.

### Study design and setting

This study is nested within Khula South Africa, an observational population-based birth cohort [40]. Pregnant women were recruited antenatally from the Gugulethu Midwife Obstetrics Unit and postnatally from nearby clinics in the area. Folic acid and iron supplementation were recommended to all mothers during pregnancy as per standard healthcare policy, according to national guidelines. Gugulethu is an urban informal settlement at sea level in the Western Cape Province and is populated by a community of primarily isiXhosa-speaking residents between 15 and 64 years of age. While approximately 70% of people are employed, close to half the population lives in informal dwellings and are at high risk of malnutrition and HIV. The majority of mother-child dyads were followed prospectively with postnatal study visits conducted at the University of Cape Town Neuroscience Institute.

### Participants

**Fig 1:**
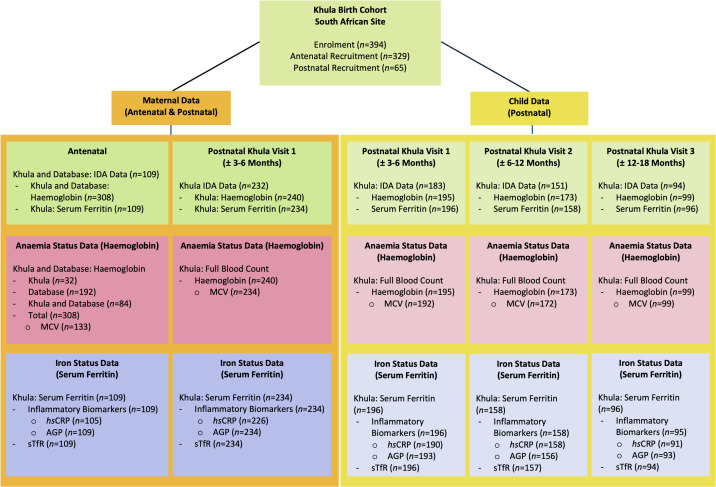
Khula Study Flow Chart Demonstrating Maternal and Child Data Available Across Timepoints for the Assessment of Iron Deficiency Anaemia (IDA) in South Africa. Abbreviations: MCV = Mean Corpuscular Volume, *hs*CRP = Highly Sensitive C-Reactive Protein, AGP = Alpha-1-Acid Glycoprotein, sTfR = Soluble Transferrin Receptor.

South African women over the age of 18 years were recruited (6^th^ December 2021 – 29^th^ November 2022) during their third trimester of pregnancy (28–40 weeks) or up to 3–6 months after childbirth. Exclusion criteria included multiple pregnancies, psychotropic drug use during pregnancy, infant congenital malformations or abnormalities (e.g., Spina Bifida, Down’s Syndrome), and severe birth complications (e.g., uterine rupture, birth asphyxia). Overall, a total of 394 mother-child pairs from South Africa were enrolled in the Khula Study, with 329 women recruited antenatally and 65 women recruited postnatally (Fig 1). IDA data including haematological metrics (haemoglobin and MCV), iron metrics (serum ferritin and sTfR), and inflammatory biomarkers (*hs*CRP and AGP) were acquired for all mothers and infants where possible. Antenatal and postnatal (first study visit; approximately 3–6 months after childbirth) IDA data was acquired for a sub-group of mothers from study samples or extracted from the National Health Laboratory Services (NHLS) database (records accessed: 8^th^ April 2024 – 12^th^ April 2024). Child IDA data was collected across three study visits postnatally (at approximately 3–6 months, 6–12 months, and 12–18 months) in early life. Maternal and infant IDA data across measures and timepoints is represented in the study flowchart (Fig 1).

### Measures

#### Contextual measures.

Demographic, psychosocial, and medical information was collected antenatally and postnatally for contextual characterisation of the sample. Self-reported maternal HIV status during pregnancy and child HIV status were confirmed by data from the Provincial Health Data Centre (PHDC), with mothers tested during pregnancy as per national policy. Antenatal maternal alcohol exposure was dichotomously classified at enrolment based on reported consumption of more than two drinks twice per week in any trimester (Alcohol Exposure Questionnaire; AEQ), or high risk of dependence in the preceding 3 months (score >12) using the Alcohol, Smoking, and Substance Involvement Screening Test (ASSIST) [41,42]. Tobacco use was also coded dichotomously based on any use reported over the previous three months using the ASSIST. For antenatally-enrolled mothers, this three-month period corresponded to the second or third trimester of pregnancy. Perinatal maternal depression was dichotomised based on moderate-severe symptomology (score >12) using the Edinburgh Postnatal Depression Scale (EPDS) [43] at enrolment. A food insecurity proxy was obtained at enrolment using items assessing food resource sufficiency in the last year (question 38 and 39) from the Life Events Questionnaire, a tool adapted from the WHO World Mental Health Survey [44]. The exacerbating effect of the COVID-19 pandemic on food insecurity was assessed using an adapted version of the COPE-IS: Coronavirus Perinatal Experiences – Impact Survey [45]. Child anthropometry was obtained by trained medical doctors on the research team at neuroimaging study visits. Child head circumference, weight, and length/height measurements were converted to z-scores based on age and sex using Anthro software for WAZ, HAZ, and HCZ. Infants were classified as underweight, stunted, or microcephalic if they had z-scores of less than -2 standard deviations.

#### Anaemia: Haematological metrics.

Antenatal maternal anaemia was assessed based on serum haemoglobin from a full blood count (FBC) in pregnancy (at Khula enrolment), or extracted from the NHLS database. For mothers with multiple measurements from either source, the lowest haemoglobin value and corresponding gestational age at the time of measurement was selected to represent the timepoint for most severe anaemia. Antenatal maternal anaemia status was dichotomously classified using recently updated (2024) WHO thresholds of <11g/dL in trimesters 1 and 3, and <10.5g/dL in trimester 2 [46]. Further severity categorisations were made indicating mild (haemoglobin 10.0 – 10.9g/dL in trimester 1 and 3; 9.5 – 10.4g/dL in trimester 2), moderate (haemoglobin 7.0 – 9.9g/dL in trimester 1 and 3; 7 – 9.4 g/dL in trimester 2), and severe (haemoglobin < 7.0g/dL for all trimesters) anaemia. For mothers for whom gestational age was not indicated at the time of measurement, haemoglobin was assigned to be from trimester 3 for classification given that this was when mothers were recruited. This is also when the physiological risk of anaemia is greatest as iron demand initially drops in pregnancy due to the cessation of menstruation, but rapidly increases with foetal growth [3,20]. Postnatal maternal anaemia was based on serum haemoglobin from an FBC at the first study visit (approximately 3–6 months after childbirth) using the WHO threshold for non-pregnant women (haemoglobin < 12g/dL) [46]. Severity was classified as mild (11 – 11.9g/dL), moderate (8 – 10.9g/dL), and severe (<8g/dL). Haemoglobin measurements were not corrected for smoking or altitude given the extremely low prevalence of maternal tobacco use and the fact that the community lives at sea level. Self-reported maternal anaemia status was recorded at enrolment for comparison with observed prevalence.

Child anaemia at study visit 1, 2, and 3 was dichotomously classified according to age-specific cut-offs using WHO guidelines [47] for children over 6 months and local guidelines for children under 6 months (Groote Schuur Hospital/University of Cape Town Pathology Laboratory; Table A in S1 Text). Classifications across timepoints were used to determine the percentage of infants diagnosed with anaemia at least once by study visit 2 (within the first 6–12 months) and study visit 3 (within the first 12–18 months).

Mean corpuscular volume (MCV), a measure of average red blood cell size and volume, was acquired from FBCs for mothers and infants. Given the widespread use of MCV in clinical practice as a proxy for anaemia aetiology, values were categorised accordingly with microcytic anaemia (below average MCV) considered likely to represent IDA, macrocytic anaemia (larger than average MCV) often being linked to pernicious anaemia (lack of limited absorption of vitamin B12) or folic acid deficiency, and normocytic anaemia (normal MCV) mostly considered to be associated with chronic infection, blood loss or kidney failure [38]. Antenatal maternal MCV measures were classified as microcytic using trimester-specific thresholds (<81µm^3^ in trimester 1 and 3, < 82 µm^3^ in trimester 2) [48]. For mothers with no gestational age at the time of measurement, MCV was assigned to trimester 3. Postnatal maternal MCV was classified as microcytic if <79µm^3^ [38]. Based on Centers for Disease Control (CDC) paediatric guidelines for the first year of life, infant MCV values were classified as microcytic if <77µm^3^ [49].

#### Inflammation: Inflammatory biomarkers.

Inflammation in mothers and infants was assessed using two well-known acute-phase response proteins, namely highly sensitive C-Reactive Protein (*hs*CRP) and Alpha-1-Acid Glycoprotein (AGP) [50]. These were used as biomarkers of inflammation (dichotomous classification) with *hs*CRP > 5mg/L and AGP > 1g/L being indicative of acute and chronic inflammation, respectively. These thresholds are consistent with WHO guidelines on interpreting serum ferritin [27,51] and BRINDA methodology for adjusting iron status [28].

#### Iron deficiency: Iron metrics.

Iron deficiency was assessed using iron metrics including serum ferritin and sTfR, with adjustment based on the inflammatory biomarkers. Serum ferritin, a protein reflecting body iron stores, was used to classify iron deficiency status before adjusting for inflammation based on WHO thresholds of <12μg/L in children under five years of age, and <15μg/L in women of reproductive age (including during pregnancy due to lack of defined thresholds for this period) [27]. However, given that serum ferritin is an acute-phase reactant known to be transiently elevated in states of inflammation, two adjustment methods were applied to obtain and compare corrected classifications of iron status. For Method A, higher adjusted WHO thresholds were used for participants with positive biomarkers of inflammation (*hs*CRP > 5mg/L or AGP > 1g/L) [27,51]. In these instances, serum ferritin concentrations of <30μg/L and <70μg/L were used to classify iron deficiency for children under five years of age and women of reproductive age, respectively. Method B was based on the BRINDA regression modelling methodology [28] using a statistical package in R (BRINDA: Computation of BRINDA Adjusted Micronutrient Biomarkers for Inflammation; R package version 0.1.5) [52] to correct continuous serum ferritin concentrations based on *hs*CRP and AGP concentrations. Standard WHO thresholds of <12μg/L and <15μg/L were subsequently used to classify iron deficiency based on the adjusted serum ferritin concentrations in infants and women of reproductive age, respectively [27].

In addition to serum ferritin, sTfR was also used as a complementary iron metric as it represents the body’s demand for iron, with the over-expression of this blood protein suggesting iron-deficient erythropoiesis. While sTfR is not an acute-phase reactant and is considered to be a stable measure for iron status in instances of inflammation [53,54], there is some evidence to suggest the need for adjustment based on AGP [29]. It is proposed that inflammation may contribute to an increase in transferrin receptor expression due to the redistribution of iron [26], resulting in the overestimation of iron-deficient erythropoiesis when AGP is high [29]. Therefore, the BRINDA regression correction approach was used to adjust sTfR concentrations in R [52]. Given that there are no standardised universal reference ranges for sTfR [51], a cutoff of >8.3mg/L was used to support a diagnosis of iron deficiency for women and infants. This was based on laboratory-specific guidelines (VitMinLab) [55] for the sandwich ELIZA technique [56] which is calibrated to commercial Ramco assay kits. This was applied to unadjusted and adjusted sTfR concentrations, and is consistent with previous BRINDA methodology in research on women and children [28].

### Statistical analysis

Sample characteristics (demographic, maternal exposures, anthropometric, and food insecurity) were presented as means and standard deviations for continuous variables and frequencies for categorical variables. All statistical analyses were conducted in SPSS (Version 29). A two-sided significance level of *p* < 0.05 was used throughout. The main analyses are described across three dimensions of classification, namely anaemia, iron deficiency, and IDA.

#### Dimension 1: Anaemia.

The first dimension of the analyses describes the profile and severity of anaemia for mothers and infants with low haemoglobin, based on WHO thresholds for classification in this cohort. It also summarises haemoglobin metrics in a population sample of South African mothers and infants without anaemia as a standard for reference. The self-reported prevalence of maternal anaemia at enrolment was recorded and the actual prevalence and severity of maternal anaemia were determined for antenatal and postnatal timepoints. Similarly, child anaemia prevalence was documented at each postnatal study visit (1–3) separately, with reporting of incidence within the first 6–12 months (by study visit 2) and 12–18 months of life (by study visit 3). To characterize the haemoglobin profile of women and infants in this population sample, the mean, standard deviation, and range of values were reported for anaemic and non-anaemic participants. Potential risk factors that may be contributing to anaemia in this context were identified by examining group differences in sociodemographic and clinical characteristics based on antenatal maternal anaemia, postnatal maternal anaemia, and child anaemia classification within the first 12–18 months of life. These were determined using independent samples t-tests for continuous data and chi-squared or Fisher’s exact tests for categorical data. Lastly, the association between antenatal and postnatal anaemia status for mothers and infants was calculated using Pearson correlation coefficients to assess whether antenatal maternal anaemia increases the risk of postnatal maternal anaemia or child anaemia.

#### Dimension 2: Iron deficiency.

The aim of the second dimension of analyses was to characterise the profile, prevalence, and severity of iron deficiency in South Africa for both anaemic and non-anaemic individuals, while accounting for inflammation. For all mothers and infants with FBCs, a descriptive report of iron metrics (serum ferritin and sTfR) and inflammatory biomarker (*hs*CRP and AGP) concentrations was provided. The prevalence of inflammation was determined for mothers and infants and, given that inflammation is known to transiently elevate serum ferritin concentrations [24,25], the associations between *hs*CRP, AGP, and unadjusted serum ferritin concentrations were explored using Pearson correlations. For both Method A (higher WHO thresholds [27]) and Method B (BRINDA corrected serum ferritin concentrations [28,29]), unadjusted and adjusted estimates of iron deficiency (based on serum ferritin) and iron-deficient erythropoiesis (based on sTfR) were represented across timepoints. For all estimates, the percentage point change was reported after accounting for inflammation. Given that the BRINDA approach adjusts serum ferritin concentrations on an individual level using corresponding inflammatory markers, it was considered to be more specific than general threshold adjustments. Therefore, sample characteristics across groups were reported based on maternal and infant iron deficiency classification with adjustment for inflammation using the BRINDA approach. Overall, this data was used to determine the extent to which iron deficiency may be underestimated in this context, and to gain insight into the necessary metrics that may be needed to make a valid diagnosis in high-risk communities.

#### Dimension 3: Iron deficiency anaemia.

For the third dimension of results, adjusted serum ferritin and MCV were used to report on the comparative prevalence and overlap between IDA and microcytic anaemia classification in anaemic mothers and infants. As the “gold standard”, overall summary statistics for mothers and infants with IDA (low haemoglobin and low serum ferritin) using BRINDA-adjusted metrics were reported. However, given the widespread clinical use of MCV as an indicator of anaemia aetiology, with microcytic anaemia recognised as a proxy for iron deficiency, this classification was reported to assess its validity as a marker of iron deficiency relative to current reference standards using BRINDA-adjusted serum ferritin. For all anaemic mothers and infants with MCV and adjusted serum ferritin data, further reporting was conducted to determine the prevalence of microcytic anaemia in this sample, and its relative overlap with IDA after adjustment for inflammation.

## Results

### Dimension 1: Anaemia

#### Antenatal maternal anaemia.

In this sample of 308 mothers (Table 1; Mean [SD] age of 28.84 [5.66] years; age range of 18 – 41.70 years), haemoglobin was available either from Khula FBCs at enrolment, or the NHLS database. Minimum haemoglobin concentrations were predominantly measured in the second (33.12% [102/308]) and third (27.92% [86/308]) trimester, at a median (IQR) gestation of 22 (14.00 - 31.50) weeks. In this sample, 34.74% (107/308) of mothers were anaemic in pregnancy, of which 63.55% (68/107) had mild anaemia, 34.58% (37/107) had moderate anaemia, and 1.87% (2/107) had severe anaemia. However, only 3.74% (4/107) of anaemic mothers correctly self-reported a positive diagnosis of anaemia during pregnancy (Table 1).

**Table 1:** Maternal and Infant Sample Characteristics According to Antenatal Maternal Anaemia Status.

Variable^a^	Total Sample (*n *= 308)		
	Maternal Anaemia (*n* = 107)	No Maternal Anaemia (*n* = 201)	*p*
**Antenatal Maternal Anaemia Profile**			
Self-reported anaemia in pregnancy ^f,g^	4 (3.74)	1 (0.50)	0.037*
Actual anaemia status in pregnancy ^b^			
Mild	68 (63.55)	n/a	
Moderate	37 (34.58)	n/a	
Severe	2 (1.87)	n/a	
Gestational age at Hb measurement (weeks) ^g^	26.70 (9.21) [4.00 – 40.00]	20.72 (9.81) [5.00 – 41.00]	<0.001***
Pregnancy trimester Hb measured ^c,g^			
First	11 (10.28)	42 (20.90)	<0.001***
Second	26 (24.30)	76 (37.81)	
Third	42 (39.25)	44 (21.89)	
Minimum Hb in pregnancy (g/dL) ^c^	9.76 (1.07) [4.40 – 10.90]	11.85 (0.74) [10.50 – 14.00]	<0.001***
Trimester 1 (*n *= 53)	10.18 (0.99) [7.90 – 10.90]	12.16 (0.70) [11.00 – 13.50]	<0.001***
Trimester 2 (*n *= 102)	9.72 (0.61) [7.70 – 10.30]	11.73 (0.77) [10.50 – 13.80]	<0.001***
Trimester 3 (*n *= 86) ^e^	9.64 (1.35) [4.40 – 10.90]	11.67 (0.59) [11.00 – 13.50]	<0.001***
**Maternal Characteristics**			
Monthly household income (ZAR) ^f,g^			
< 1000	20 (18.69)	34 (16.92)	0.060
1000-5000	57 (53.27)	82 (40.80)	
5000-10000	22 (20.56)	51 (25.37)	
> 10000	1 (0.93)	12 (5.97)	
Education ^f^			
Primary	2 (1.87)	4 (1.99)	0.609
Some secondary	55 (51.40)	84 (41.79)	
Completed secondary	37 (34.58)	84 (41.79)	
Some tertiary	8 (7.48)	16 (7.96)	
Completed tertiary	5 (4.67)	13 (6.47)	
Employed ^g^	34 (31.78)	74 (36.82)	0.361
Age at enrolment (years) ^g^	28.93 (5.65) [18.50 – 40.50]	28.79 (5.69) [18.00 – 41.70]	0.843
Food insecurity	55 (51.40)	119 (59.20)	0.188
COVID-19 effect on food insecurity ^g^			
No disruption	37 (34.58)	85 (42.29)	0.470
Some disruption	12 (11.21)	34 (16.92)	
Extreme disruption	17 (15.89)	28 (13.93)	
Smoking during pregnancy ^f^	7 (6.54)	5 (2.49)	0.119
Alcohol during pregnancy	10 (9.35)	9 (4.48)	0.091
Depression during pregnancy	18 (16.82)	34 (16.92)	0.983
HIV infection during pregnancy ^g^	47 (43.93)	57 (28.36)	0.005**
**Infant Characteristics at Birth**			
Sex (male) ^g^	37 (34.58)	93 (46.27)	0.249
Gestational age at birth (weeks) ^g^	38.85 (1.87) [32.00 – 42.00]	39.22 (1.86) [30.00 – 42.00]	0.145
HIV infection ^f,g^	1 (0.93)	0 (0.00)	0.439
Birth weight (g) ^d,g^	3104.93 (484.49) [1950.00 – 4360.00]	3222.01 (504.89) [1180.00 – 4750.00]	0.077
Birth length (cm) ^d,g^	49.33 (3.46) [41.00 – 57.00]	50.14 (3.40) [33.00 – 57.00]	0.075
Birth head circumference (cm) ^d,g^	34.58 (1.58) [30.50 – 38.00]	34.65 (1.79) [28.00 – 40.00]	0.783

Abbreviations: Hb, haemoglobin; ZAR, South African Rand; HIV, Human Immunodeficiency Virus; COVID-19, Coronavirus Disease 2019; g, grams; cm, centimetres.

SI conversion factor: To convert to haemoglobin grams per litre, multiply by 10.

^a^Values for continuous variables are presented as: mean (standard deviation) [range]. Values for categorical variables are presented as: count (%).

^b^Maternal anaemia during pregnancy was classified according to the WHO thresholds of <11g/dL for trimesters 1 and 3, and <10.5g/dL for trimester 2. Severity categorisations were made indicating mild (haemoglobin 10.0 – 10.9g/dL in trimester 1 and 3; 9.5-10.4 in trimester 2), moderate (haemoglobin 7.0 – 9.9g/dL in trimester 1 and 3; 7 – 9.4 in trimester 2), and severe (haemoglobin < 7.0g/dL for all trimesters) anaemia categories.

^c^Trimester of pregnancy defined as first (0–12 weeks), second (13–27 weeks), and third (28 weeks onwards).

^d^The birth anthropometric measurements were conducted by trained labour staff in the ward. Infant length was measures in cm to the nearest completed 0.5 cm and weight was measured in kgs.

^e^Levene’s test was significant. *T*-test results were interpreted based on equal variance not assumed.

^f^Fisher’s exact test result interpreted due to one or more cells having an expected count of less than 5.

^g^Missing values: self-reported anaemia in pregnancy (*n* = 44), gestational age at maternal minimum haemoglobin measurement (*n* = 67), pregnancy trimester Hb measured (*n* = 67), monthly household income (*n* = 29), maternal employment (*n* = 1), maternal age at enrolment (*n* = 67), COVID-19 effect on food insecurity (*n* = 95), maternal HIV infection during pregnancy (*n* = 5), infant sex (*n* = 44), infant gestational age at birth (*n* = 67), infant HIV infection *(n* = 226), infant birth weight (*n* = 46), infant birth length (*n* = 50), infant birth head circumference (*n* = 49).

*p is significant at <0.05, ** *p* is significant at <0.01, ***p is significant at <0.001.

There were no group differences in maternal and infant sample characteristics between anaemic and non-anaemic mothers, with the exception of maternal HIV infection which was significantly more prevalent in mothers with anaemia (43.93% [47/107]) than mothers without anaemia (28.36% [57/201]) in pregnancy. While smoking and alcohol use in pregnancy were not common, the prevalence of reported food insecurity was high in both mothers with antenatal anaemia (51.40% [55/107]) and mothers without (59.20% [119/201]). Additionally, the effect of the COVID-19 pandemic on food insecurity was similar with approximately one third of both groups reporting some or extreme disruption.

#### Postnatal maternal anaemia.

At study visit 1 (approximately 3–6 months after childbirth), 240 mothers (mean [SD] age of 28.95 [5.59] years; range of 18 – 44 years) had haemoglobin measures from Khula FBCs (Table 2). Overall, 22.50% (54/240) of mothers were found to be anaemic postnatally, of which 75.93% (41/54) had mild anaemia, 20.37% (11/54) had moderate anaemia, and 3.70% (2/54) had severe anaemia. As seen in the group of mothers with antenatal maternal haemoglobin data, there were no group differences in maternal or infant sample characteristics at study visit 1, with the exception of maternal HIV infection which was more prevalent in mothers with postnatal anaemia (50% [27/54]) than mothers without postnatal anaemia (31.18% [58/186]; Table 2).

**Table 2:** Maternal and Infant Sample Characteristics According to Postnatal Maternal Anaemia Status at Study Visit 1 (±3-6 Months after Childbirth).

Variable^a^	Total Sample (*n *= 240)		
	Maternal Anaemia (*n* = 54)	No Maternal Anaemia (*n* = 186)	*p*
**Postnatal Maternal Anaemia Profile**			
Anaemia status ^b^			
Mild	41 (75.93)	n/a	
Moderate	11 (20.37)	n/a	
Severe	2 (3.70)	n/a	
Hb concentration (g/dL)	11.16 (0.85) [7.30 – 11.90]	13.11 (0.74) [12.00 – 15.00]	<0.001***
**Maternal Characteristics**			
Monthly household income (ZAR) ^d,e^			
< 1000	10 (18.52)	35 (18.82)	0.204
1000-5000	28 (51.85)	78 (41.94)	
5000-10000	15 (27.78)	43 (23.12)	
> 10000	0 (0.00)	12 (6.45)	
Education ^d^			
Primary	1 (1.85)	5 (2.69)	0.413
Some secondary	29 (53.70)	84 (45.16)	
Completed secondary	20 (37.04)	71 (38.17)	
Some tertiary	1 (1.85)	17 (9.14)	
Completed tertiary	3 (5.56)	9 (4.84)	
Employed ^e^	18 (33.33)	64 (34.41)	0.844
Age at study visit 1 (years)	29.31 (5.71) [18.00 – 42.00]	28.84 (5.56) [18.00 – 44.00]	0.587
Food insecurity	30 (55.56)	102 (54.84)	0.926
COVID-19 effect on food insecurity ^e^			
No disruption	31 (57.41)	103 (55.38)	0.190
Some disruption	8 (14.81)	45 (24.19)	
Extreme disruption	14 (25.93)	32 (17.20)	
Smoking at enrolment ^d^	2 (3.70)	7 (3.76)	1
Alcohol at enrolment ^d^	3 (5.56)	10 (5.38)	1
Depression at enrolment	10 (18.52)	30 (16.13)	0.678
HIV infection at enrolment	27 (50.00)	58 (31.18)	0.011*
**Infant Characteristics at Study Visit 1**			
Age (months) ^e^	3.64 (0.66) [2.37 – 5.03]	3.82 (0.81) [2.01 – 5.85]	0.188
Sex (male)	29 (53.70)	96 (51.61)	0.787
HIV infection ^e^	0 (0.00)	0 (0.00)	n/a
Microcephaly ^c,d,e^	0 (0.00)	1 (0.54)	1
Underweight ^c,d,e^	3 (5.56)	4 (2.15)	0.169
Stunting ^c,e^	7 (12.96)	24 (12.90)	0.892

Abbreviations: Hb, haemoglobin; ZAR, South African Rand; HIV, Human Immunodeficiency Virus; COVID-19, Coronavirus Disease 2019; g, grams; cm, centimetres.

SI conversion factor: To convert to haemoglobin grams per litre, multiply by 10.

^a^Values for continuous variables are presented as: mean (standard deviation) [range]. Values for categorical variables are presented as: count (%).

^b^Postnatal maternal anaemia was classified according to the WHO threshold of <12g/dL for women of reproductive age. Severity classifications were defined as mild (11-11.9g/dL), moderate (8-10.9g/dL), and severe (<8g/dL).

^c^The anthropometric measurements at timepoint 1 were conducted by trained medical staff on the research team. Child weight, length, and head circumference measurements were converted to z-scores based on age and sex using Anthro software for WAZ, HAZ, and HCZ. Infants were classified as underweight, stunted, or having microcephaly if they had z-scores of less than -2 SDs.

^d^Fisher’s exact test result interpreted due to one or more cells having an expected count of less than 5.

^e^Missing values: monthly household income (*n* = 19), maternal employment (*n* = 2), COVID-19 effect on food insecurity *(n* = 7), infant age at study visit 1 (*n* = 52), infant HIV infection (*n* = 165), infant microcephaly at study visit 1 (*n* = 6), infant underweight at study visit 1 (*n* = 6), infant stunting at study visit 1 (*n* = 9).

*p is significant at <0.05, **p is significant at <0.01, ***p is significant at <0.001.

There was overlap between samples across timepoints with 85% (204/240) of mothers with haemoglobin data at postnatal study visit 1, also having antenatal data as described in the previous section. Antenatal maternal anaemia status and postnatal maternal anaemia status were significantly positively associated (*n *= 204), *r *= 0.3, *p *< 0.001.

#### Child anaemia.

Child haemoglobin was available from FBCs across the first year of life at study visit 1 (*n* = 195; Mean [SD] age of 3.79 [0.77] months, age range of 2 – 5.85 months), study visit 2 (*n* = 173; Mean [SD] of 8.54 [1.59] months, age range of 5.29 – 12.20 months), and study visit 3 (*n* = 99; Mean [SD] age of 14.16 [1.25], age range of 12 – 18.05 months). Of these infants (Table 3), the prevalence of child anaemia was 51.28% (100/195), 27.17% (47/173), and 20.20% (20/99), respectively. Overall, 46.82% (125/267) of infants were classified as anaemic at least once in the first 6–12 months of life (by study visit 2) and 48.10% (136/283) were classified as anaemic at least once in the first 12–18 months of life (by study visit 3). Risk factors for child anaemia in this sample included being underweight (6% of anaemic infants versus 0% of non-anaemic infants at study visit 1), antenatal alcohol exposure (12.77% of anaemic infants versus 2.38% of non-anaemic infants at study visit 2), and antenatal maternal anaemia (55% of anaemic infants versus 31.65% of non-anaemic infants at study visit 3).

**Table 3:** Maternal and Infant Sample Characteristics According to Child Anaemia Status Across Study Visits.

Variable ^a^	Study Visit 1 (±3–6 months; *n *= 195)			Study Visit 2 (±6–12 months; *n* = 173)			Study Visit 3 (±12–18 months; *n* = 99)		
	Child Anaemia ^b^ (*n *= 100)	No Child Anaemia (*n *= 95)	*p*	Child Anaemia ^b^ (*n *= 47)	No Child Anaemia (*n *= 126)	*p*	Child Anaemia ^b^ (*n *= 20)	No Child Anaemia (*n *= 79)	*p*
**Infant Characteristics**									
Hb (g/dL)	10.18 (0.68) [7.40 – 11.00]	11.40 (0.79) [9.50 – 14.10]	<0.001***	9.94 (0.55) [8.00 – 10.60]	11.65 (0.83) [10.50 – 15.30]	<0.001***^f^	9.69 (0.85) [7.30 – 10.40]	11.41 (0.67) [10.50 – 13.10]	<0.001***
Age (months)	3.86 (0.72) [2.17 – 5.85]	3.71 (0.82) [2.01 – 5.49]	0.168	8.29 (1.67) [5.29 – 11.87]	8.63 (1.55) [5.29 – 12.20]	0.216	14.46 (1.05) [12.76 – 17.06]	14.09 (1.29) [12.00 – 18.05]	0.236
Sex (male)	50 (50.00)	50 (52.63)	0.713	24 (51.06)	60 (47.62)	0.687	9 (45.00)	50 (63.29)	0.136
HIV infection	0 (0.00)	0 (0.00)	n/a^e^	0 (0.00)	1 (0.79)	1 ^d,e^	1 (5.00)	0 (0.00)	0.262^d,e^
Microcephaly ^c^	2 (2.00)	0 (0.00)	0.498 ^d,e^	0 (0.00)	4 (3.17)	0.576 ^d^	0 (0.00)	1 (1.27)	1 ^d^
Underweight ^c^	6 (6.00)	0 (0.00)	0.029* ^d,e^	0 (0.00)	5 (3.97)	0.325 ^d^	1 (5.00)	1 (1.27)	0.365 ^d^
Stunting ^c^	15 (15.00)	7 (7.37)	0.103^e^	5 (10.64)	9 (7.14)	0.530 ^d,e^	4 (20.00)	8 (10.13)	0.255 ^d^
**Maternal Characteristics**									
Antenatal maternal anaemia ^b^	26 (26.00)	26 (27.37)	0.795^e^	16 (34.04)	31 (24.60)	0.077^e^	11 (55.00)	25 (31.65)	0.013* ^e^
Postnatal maternal anaemia ^b^	28 (28.00)	17 (17.89)	0.102^e^	13 (27.66)	20 (15.87)	0.117^e^	6 (30.00)	13 (16.46)	0.091 ^d,e^
Monthly household income (ZAR)									
< 1000	20 (21.00)	20 (21.05)	0.386 ^d,e^	6 (12.77)	21 (16.67)	0.497 ^d,e^	4 (20.00)	19 (24.05)	0.871 ^d,e^
1000-5000	47 (47.00)	35 (36.84)		28 (59.57)	59 (46.83)		10 (50.00)	40 (50.63)	
5000-10000	21 (21.00)	29 (30.53)		8 (17.02)	29 (23.02)		4 (20.00)	12 (15.19)	
> 10000	4 (4.00)	3 (3.16)		1 (2.13)	7 (5.56)		0 (0.00)	4 (5.06)	
Education									
Primary	2 (2.00)	1 (1.05)	0.988 ^d^	1 (2.13)	2 (1.59)	0.112 ^d^	0 (0.00)	3 (3.80)	0.893 ^d^
Some secondary	48 (48.00)	47 (49.47)		19 (40.43)	61 (48.41)		12 (60.00)	36 (45.57)	
Completed secondary	38 (38.00)	35 (36.84)		24 (51.06)	43 (34.13)		6 (30.00)	30 (37.97)	
Some tertiary	7 (7.00)	8 (8.42)		1 (2.13)	15 (11.90)		1 (5.00)	6 (7.59)	
Completed tertiary	5 (5.00)	4 (4.21)		2 (4.26)	5 (3.97)		1 (5.00)	4 (5.06)	
Employed	30 (30.00)	37 (38.95)	0.206^e^	16 (34.04)	40 (31.75)	0.707^e^	5 (25.00)	31 (39.24)	0.222 ^e^
Age at enrolment (years)	28.78 (5.57) [18.00 – 40.70]	29.18 (5.73) [19.40 – 42.60]	0.616	29.64 (6.11) [18.10 – 41.00]	28.76 (5.52) [18.90 – 42.60]	0.370	29.17 (6.10) [18.90 – 39.50]	29.67 (6.02) [18.00 – 44.10]	0.739
Food insecurity	58 (58.00)	50 (52.63)	0.451	29 (61.70)	71 (56.35)	0.526	9 (45.00)	48 (60.76)	0.203
COVID-19 effect on food insecurity									
No disruption	61 (61.00)	47 (49.47)	0.175^e^	17 (36.17)	54 (42.86)	0.228^e^	7 (35.00)	32 (40.51)	0.514 ^d,e^
Some disruption	18 (18.00)	22 (23.16)		11 (23.40)	18 (14.29)		2 (10.00)	14 (17.72)	
Extreme disruption	15 (15.00)	22 (23.16)		11 (23.40)	18 (14.29)		6 (30.00)	15 (18.99)	
Smoking during pregnancy	4 (4.00)	3 (3.16)	1 ^d^	1 (2.13)	5 (3.97)	1 ^d^	1 (5.00)	6 (7.59)	1 ^d^
Alcohol during pregnancy	5 (5.00)	3 (3.16)	0.722 ^d^	6 (12.77)	3 (2.38)	0.013* ^d^	2 (10.00)	7 (8.86)	1 ^d^
Depression during pregnancy	13 (13.00)	19 (20.00)	0.187	7 (14.89)	22 (17.46)	0.688	3 (15.00)	13 (16.46)	1^d^
HIV infection	36 (36.00)	31 (32.63)	0.621	14 (29.79)	50 (39.68)	0.230	12 (60.00)	33 (41.77)	0.144

Abbreviations: Hb, haemoglobin; ZAR, South African Rand; HIV, Human Immunodeficiency Virus; COVID-19, Coronavirus Disease 2019; g, grams; cm, centimetres.

SI conversion factor: To convert to haemoglobin grams per litre, multiply by 10.

^a^Values for continuous variables are presented as: mean (standard deviation) [range]. Values for categorical variables are presented as: count (%).

^b^Maternal anaemia during pregnancy was classified according to the WHO thresholds of <11g/dL for trimesters 1 and 3, and <10.5g/dL for trimester 2. Postnatal maternal anaemia was classified according to the WHO threshold of <12g/dL for women of reproductive age. Child anaemia was classified using age-specific WHO thresholds for children over 6 months and local guidelines for children under 6 months.

^c^The anthropometric measurements were conducted by trained medical staff on the research team. Child weight, length, and head circumference measurements were converted to z-scores based on age and sex using Anthro software for WAZ, HAZ, and HCZ. Infants were classified as underweight, stunted, or having microcephaly if they had z-scores of less than -2 SDs.

^d^Fisher’s exact test result interpreted due to one or more cells having an expected count of less than 5.

^e^Missing values: infant HIV infection (*n* = 136 at study visit 1, *n* = 116 at study visit 2, *n* = 57 at study visit 3), infant microcephaly (*n* = 4 at study visit 1), infant underweight (*n* = 4 at study visit 1), infant stunting (*n* = 6 at study visit 1, *n* = 2 at study visit 2), antenatal maternal anaemia status (*n* = 28 at study visit 1, *n* = 37 at study visit 2, *n *= 18 at study visit 3), postnatal maternal anaemia status (*n* = 7 at study visit 1, *n* = 48 at study visit 2, *n* = 27 at study visit 3), monthly household income (*n *= 16 at study visit 1, *n* = 14 at study visit 2, *n* = 6 at study visit 3), maternal employment (*n *= 1 at study visit 1, *n *= 1 at study visit 2, *n *= 1 at study visit 3), COVID-19 effect on food insecurity (*n* = 10 at study visit 1, *n* = 44 at study visit 2, *n* = 23 at study visit 3).

fLevene’s test was significant. *T*-test results were interpreted based on equal variance not assumed.

*p is significant at <0.05, ** *p* is significant at <0.01, ***p is significant at <0.001.

While antenatal maternal anaemia status was not significantly correlated with child anaemia status at visit 1 (*n *= 167, *r *= -0.20, *p *= 0.796) or study visit 2 (*n *= 136, *r *= 0.152, *p *= 0.078), a significantly positive association was observed at study visit 3 (*n *= 81, *r *= 0.277, *p *= 0.012). Postnatal maternal anaemia status was not significantly correlated with child anaemia at study visit 1 (*n *= 188, *r* = 0.119, *p* = 0.103), or with child anaemia at later timepoints: study visit 2 (*n *= 125, *r* = 0.140, *p* = 0.119), or study visit 3 (*n *= 72, *r *= 0.211, *p *= 0.076).

### Dimension 2: Iron deficiency

#### Biomarkers of inflammation.

For all mothers and infants with serum ferritin data available, inflammatory biomarkers were used as indicators of inflammation. Overall, *hs*CRP and AGP concentrations were positively correlated with unadjusted serum ferritin concentrations for mothers and infants across study visits (Table 4). This link between inflammation and raised serum ferritin was found to be significant for *hs*CRP in mothers postnatally and infants at study visit 2, and AGP in mothers (antenatally and postnatally) and infants at study visit 2 and study visit 3.

**Table 4:** Associations between Inflammatory Biomarker Concentrations and Unadjusted Serum Ferritin Concentrations In Mothers and Infants Across Study Visits.

Inflammatory Biomarker	Unadjusted Serum Ferritin				
	Antenatal Maternal ^a^ (*n *= 109)	Postnatal Maternal ^a^ (*n *= 234)	Child Visit 1 ^a^ (*n *= 196)	Child Visit 2 ^a^ (*n *= 158)	Child Visit 3 ^a^ (*n *= 96)
***hs*CRP**	0.117	0.400***	0.076	0.188*	0.057
**AGP**	0.453***	0.367***	0.025	0.294***	0.371***

Abbreviations*. hs*CRP = highly sensitive C-Reactive Protein, AGP = Alpha-1-Acid Glycoprotein.

^a^Missing values: Antenatal Maternal *hs*CRP (*n *= 4), postnatal maternal *hs*CRP (*n *= 8), infant *hs*CRP (*n *= 6 at study visit 1, *n *= 5 at study visit 3), infant AGP (*n *= 3 at study visit 1, *n *= 2 at study visit 2, *n *= 3 at study visit 3).

*p is significant at <0.05, **p is significant at <0.01, ***p is significant at <0.001.

As per WHO guidelines [27,51], *hs*CRP and AGP concentrations (descriptive statistics for the full group presented in Supplementary Information; Table B in S1 Text) were used to assess the prevalence of acute (*hs*CRP > 5mg/L) and chronic inflammation (AGP > 1g/L) status for mothers and infants across study visits (Table 5). Overall, 46.79% (51/109) of mothers had evidence of inflammation in pregnancy, most of which was acute (raised *hs*CRP; 44.95% [49/109]) rather than chronic (raised AGP; 3.67% [4/109]). The prevalence of postnatal maternal inflammation remained high (51.28% [120/234]), with evidence for both acute (raised *hs*CRP; 28.63% [67/234]) and chronic inflammation (raised AGP; 42.74% [100/234]). In infants, the prevalence of inflammation increased (19.90% [39/196], 36.08% [57/158], and 42.71% [41/96] at study visits 1–3, respectively), with higher biomarkers for chronic inflammation (AGP > 1g/L).

**Table 5:** Estimated Iron Deficiency and Iron-Deficient Erythropoiesis Status of Mothers and Infants With and Without Adjustment for Inflammation Across Study Visits.

Variable ^a^	Inflammatory Status			Serum Ferritin Concentration (μg/L)		Iron Deficiency Status Classification By Serum Ferritin			Soluble Transferrin Receptor (sTfR) Concentration (mg/L)		Iron-Deficient Erythropoiesis Classified By sTfR	
	Acute (*hs*CRP > 5mg/L)	Chronic (AGP > 1g/L)	Overall (*hs*CRP > 5mg/L or AGP > 1g/L)	Unadjusted	Adjusted: BRINDA Regression ^b^	Unadjusted ^c^	Adjusted		Unadjusted	Adjusted ^e^	Unadjusted ^f^	Adjusted: BRINDA Regression ^f^
							Method A: Higher WHO Thresholds ^d^	Method B: BRINDA Regression ^c^				
**Antenatal Maternal** **(*n *= 109)**	49 (44.95) ^g^	4 (3.67)	51 (46.79)	38.65 (38.32) [8.79 – 245.60]	25.70 (21.87) [5.47 – 139.28]	20 (18.35)	60 (55.04)	42 (38.53)	5.05 (1.99) [1.73 – 14.05]	4.84 (1.85) [1.73 – 12.39]	6 (5.50)	6 (5.50)
**Postnatal Maternal** **(*n *= 234)**	67 (28.63) ^g^	100 (42.74)	120 (51.28)	47.63 (39.34) [4.12 – 278.60]	29.25 (21.00) [3.14 – 115.53]	28 (11.97)	109 (46.58)	59 (25.21)	5.82 (2.17) [1.78 – 15.70]	4.07 (1.24) [1.78 – 12.01]	25 (10.68)	1 (0.43)
**Child** **Visit 1** **(*n *= 196)**	21 (10.71) ^g^	34 (17.35)^g^	39 (19.90)	101.49 (66.82) [9.41 – 345.80]	93.14 (61.28) [8.63 – 305.66]	1 (0.51)	3 (1.53)	1 (0.51)	6.59 (1.66) [3.71 – 15.83]	6.35 (1.54) [3.71 – 15.83]	23 (11.73)	15 (7.65)
**Child** **Visit 2** **(*n *= 158)**	24 (15.19)	51 (32.28) ^g^	57 (36.08)	44.95 (33.33) [6.98 – 278.5]	36.29 (25.78) [6.44 – 200.71]	6 (3.80)	24 (15.19)	10 (6.33)	7.47 (2.03) [4.79 – 19.04]	7.01 (1.80) [4.17 – 17.43]	38 (24.05) ^g^	24 (15.19) ^g^
**Child** **Visit 3** **(*n *= 96)**	14 (14.58) ^g^	40 (41.67) ^g^	41 (42.71)	35.05 (33.88) [4.01 – 246.5]	22.45 (18.12) [2.55 – 107.59]	19 (19.79)	30 (31.25) ^g^	31 (32.29) ^g^	7.73 (3.43) [1.84 – 27.5]	6.14 (2.39) [1.84 – 21.12]	29 (30.21) ^g^	10 (10.42) ^g^

Abbreviations. *hs*CRP = highly sensitive C-Reactive Protein, AGP = Alpha-1-Acid Glycoprotein; sTfR, soluble transferrin receptor.

^a^Values for continuous variables are presented as: mean (standard deviation) [range]. Values for categorical variables are presented as: count (%).

^b^Serum ferritin concentrations were adjusted for inflammation using both *hs*CRP and AGP preferentially, and only *hs*CRP or AGP where both were not available. Adjusted serum ferritin concentrations for women of reproductive age and pre-school age children were computed according to the BRINDA regression approach using a statistical package in R.

^c^Unadjusted iron deficiency status and adjusted iron deficiency status using Method B (BRINDA approach) was classified using the WHO thresholds for serum ferritin of <12μg/L in children under five years of age, and <15 μg/L in women (including during pregnancy).

^d^To classify iron deficiency using adjustment Method A for serum ferritin, adjusted iron deficiency status was classified using higher adjusted WHO thresholds for participants with positive biomarkers of inflammation (*hs*CRP > 5mg/L or AGP > 1g/L). For mothers and infants with no inflammation, unadjusted serum ferritin concentrations were classified using the standard WHO thresholds of <15μg/L and <12μg/L, respectively. For mothers and infants with inflammation, unadjusted serum ferritin concentrations were classified using adjusted WHO thresholds of <70μg/L and <30 μg/L, respectively.

^e^sTfR concentrations were adjusted for inflammation using AGP only. Adjusted sTfR for women of reproductive age and children of preschool age were computed according to the BRINDA regression approach using a statistical package in R.

^f^Unadjusted and adjusted sTfR concentrations >8.3mg/L were classified iron-deficient erythropoiesis.

^g^Missing values: antenatal maternal *hs*CRP (*n* = 4), postnatal maternal *hs*CRP (*n* = 8), infant *hs*CRP (*n* = 6 at study visit 1, *n* = 5 at study visit 3), infant AGP (*n* = 3 at study visit 1, *n* = 2 at study visit 2, *n* = 3 at study visit 3), infant overall infection (*n* = 1 at study visit 3), Method A infant adjusted iron deficiency status (*n *= 1 at study visit 3), Method B infant adjusted iron deficiency status (*n* = 1 at study visit 3), unadjusted infant sTfR (*n* = 1 at study visit 2, *n* = 2 at study visit 3), adjusted infant sTfR (*n* = 3 at study visit 2, *n* = 4 at study visit 3).

#### Antenatal maternal iron deficiency.

**Fig 2:**
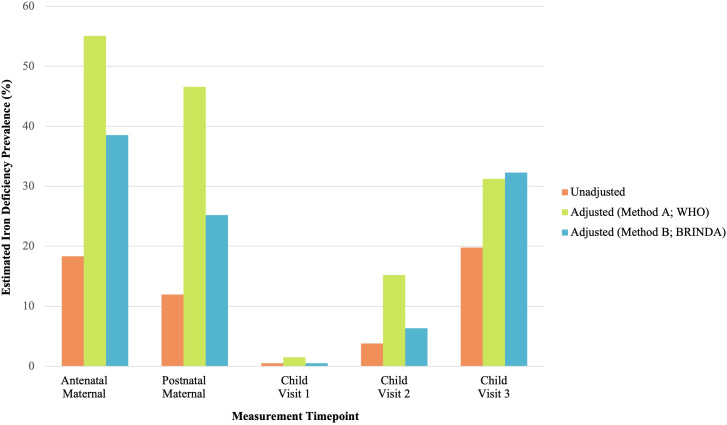
Estimated Prevalence of Iron Deficiency After Adjustment for Inflammation Using Two Methods in Mothers and Infants Across Study Visits.

In this study (Table 5), a sample of 109 mothers (Mean [SD] age at enrolment: 28.75 [5.75] years; range: 19 – 40.4 years) had antenatal serum ferritin available from Khula enrolment in pregnancy. The prevalence of antenatal maternal iron deficiency (serum ferritin < 15μg/L) using unadjusted serum ferritin concentrations was 18.35% (20/109). However, adjusting for inflammation using Methods A and B increased the prevalence to 55.04% (60/109; 36.69 percentage point increase) and 38.53% (42/109; 20.18 percentage point increase), respectively (Fig 2). Adjusting sTfR concentrations using the BRINDA approach made no difference to the estimates of iron-deficient erythropoiesis (high sTfR) due to very few (*n *= 4) of the mothers having raised AGP.

#### Postnatal maternal iron deficiency.

At the first study visit 1 (approximately 3–6 months after childbirth), 234 mothers (Mean [SD] age at study visit 1: 28.94 [5.58] years; range: 18 – 44 years) had serum ferritin available from postnatal FBCs (Table 5). The prevalence of postnatal maternal iron deficiency (serum ferritin < 15μg/L) using unadjusted serum ferritin concentrations was 11.97% (28/234). However, adjusting for inflammation using Methods A and B increased the prevalence to 46.58% (109/234; 34.61 percentage point increase) and 25.21% (59/234; 13.24 percentage point increase), respectively (Fig 2). Adjusting sTfR concentrations based on AGP using the BRINDA approach decreased the estimated prevalence of iron-deficient erythropoiesis from 10.68% (25/234) to 0.43% (1/234; 10.25 percentage point decrease).

Overall, iron deficiency prevalence was consistently lower postnatally than antenatally, based on unadjusted and adjusted estimates. However, the prevalence of iron deficiency in mothers, both during and after pregnancy, was underestimated when inflammatory biomarkers (*hs*CRP and AGP) were unaccounted for (Fig 2). In contrast, the estimated prevalence of iron-deficient erythropoiesis was overestimated postnatally in mothers with chronic inflammation (raised AGP) after childbirth.

#### Child iron deficiency.

At study visit 1, 196 infants (Mean [SD] age: 3.77 [0.77] months; range: 2 – 5.85 months) had serum ferritin available (Table 5). The prevalence of child iron deficiency (serum ferritin < 12μg/L) using unadjusted serum ferritin concentrations was 0.51% (1/196). Adjustment for inflammation using Method A only marginally increased the prevalence to 1.53% (3/196; 1.02 percentage point increase). No change in prevalence was observed using adjustment Method B (Fig 2). Adjusting sTfR concentrations based on AGP using the BRINDA approach decreased the estimated prevalence of iron-deficient erythropoiesis from 11.73% (23/196) to 7.65% (15/196; 4.08 percentage point decrease).

At study visit 2, 158 infants (Mean [SD] age: 8.53 [1.59] months; range: 5.29 – 12.20 months) had serum ferritin available (Table 5). The prevalence of child iron deficiency (serum ferritin < 12μg/L) using unadjusted serum ferritin concentrations was 3.80% (6/158). Adjustment for inflammation using Methods A and B marginally increased the prevalence to 15.19% (24/158; 11.39 percentage point increase) and 6.33% (10/158; 2.53 percentage point increase), respectively (Fig 2). Adjusting sTfR concentrations based on AGP using the BRINDA approach decreased the estimated prevalence of iron-deficient erythropoiesis from 24.05% (38/158) to 15.19% (24/158; 8.86 percentage point decrease).

At study visit 3, 96 infants (Mean [SD] age: 14.17 [1.26] months; range: 12 – 18.05 months) had serum ferritin available (Table 5). The prevalence of child iron deficiency (serum ferritin < 12μg/L) using unadjusted serum ferritin concentrations was 19.79% (19/96). Adjustment for inflammation increased the prevalence of estimated iron deficiency to 31.25% (30/96; 11.46 percentage point increase) and 32.29% (31/96; 12.5% increase) for Methods A and B, respectively (Fig 2). Adjusting sTfR concentrations based on AGP using the BRINDA approach decreased the estimated prevalence of iron-deficient erythropoiesis from 30.21% (29/96) to 10.42% (10/96; 19.79 percentage point decrease).

Overall, the prevalence of iron deficiency in childhood was relatively low at study visits 1 and 2, but increased with age across the first 12–18 months of life. This trajectory was more prominent once inflammation (*hs*CRP and AGP) had been accounted for, with unadjusted serum ferritin underestimating the prevalence of child iron deficiency (Fig 2). Similarly, the estimated prevalence of iron-deficient erythropoiesis was consistently overestimated when not adjusted for chronic inflammation (AGP), particularly by study visit 3.

#### Sample characteristics by iron deficiency status.

Sample characteristics across groups for mothers and infants were reported based on iron deficiency classification with adjustment for inflammation using BRINDA, which is likely to be more specific (Supplementary Information, Tables C, D, E in S1 Text). Group differences were not assessed for infants at study visit 1 given that only one child was found to be iron deficient at this timepoint. Overall, after accounting for inflammation, adjusted serum ferritin concentrations were significantly lower in mothers and infants with iron deficiency across timepoints. Similarly, adjusted sTfR concentrations were significantly higher, suggestive of iron-deficient erythropoiesis in mothers and infants with iron deficiency across timepoints. Maternal and infant characteristics were similar between groups.

### Dimension 3: Iron deficiency anaemia

In a sub-group of anaemic mothers and infants with both adjusted serum ferritin data and MCV data, the prevalence of IDA was reported using anaemia classifications and adjusted iron deficiency classifications from the BRINDA approach (Method B; Table 6). Iron deficiency was found to account for approximately half of anaemia cases (Table 6) in mothers antenatally (50% [20/40]) and postnatally (45.10% [23/51]), and in infants at 12–18 months postnatally (55.56% [10/18]). The prevalence of IDA in infants at 3–6 months (1.11% [1/90]) and 6–12 months (11.36% [5/44]) was low, indicating that anaemia at these timepoints may not be due to iron deficiency.

**Table 6:** Iron Deficiency and Microcytic Anaemia Classification Using Adjusted Serum Ferritin and MCV Data for Mothers and Infants Across Study Visits.

Anaemia Classification ^a,b^	Adjusted Serum Ferritin (μg/L)	IDA Prevalence ^c^	MCV (µm^3^)	Microcytic Anaemic Prevalence ^d^	Overlap: IDA and Microcytic Anaemia
**Antenatal Maternal** **(*n *= 40)**	25.43 (26.28) [6.25 – 139.28]	20 (50.00)	87.98 (6.08) [71.00 – 99.00]	3 (7.50)	2 (5.00)
**Postnatal Maternal** **(*n *= 51)**	19.82 (17.85) [3.14 – 115.53]	23 (45.10)	87.10 (7.43) [71.00 – 109.00]	7 (13.73)	4 (7.84)
**Child Visit 1** **(*n *= 90)**	104.04 (68.13) [8.63 – 305.66]	1 (1.11)	81.60 (4.98) [69.00 – 92.00]	17 (18.89)	1 (1.11)
**Child Visit 2** **(*n *= 44)**	31.25 (20.39) [6.44 – 102.99]	5 (11.36)	75.43 (4.83) [63.00 – 85.00]	27 (61.36)	5 (11.36)
**Child Visit 3** **(*n *= 18)**	13.50 (9.69) [2.55 – 42.62]	10 (55.56)	73.28 (5.15) [65.00 – 84.00]	12 (66.67)	8 (44.44)

Abbreviations: IDA, iron deficiency anaemia; MCV, mean corpuscular volume.

^a^Values for continuous variables are presented as: mean (standard deviation) [range]. Values for categorical variables are presented as: count (%).

^b^Maternal anaemia during pregnancy was classified according to the WHO thresholds of <11g/dL for trimesters 1 and 3, and <10.5g/dL for trimester 2. Postnatal maternal anaemia was classified according to the WHO threshold of <12g/dL for women of reproductive age. Child anaemia was classified using age-specific WHO thresholds for children over 6 months and local guidelines for children under 6 months.

^c^Iron deficiency anaemia was classified for mothers and infants with anaemia (low haemoglobin) and iron deficiency (low serum ferritin). Iron deficiency was classified using adjustment Method B for serum ferritin whereby unadjusted serum ferritin concentrations were adjusted for inflammation according to the BRINDA regression modelling approach using a statistical package in R. Iron deficiency status was classified using adjusted serum ferritin concentrations and the standard WHO thresholds of <15 μg/L and <12 μg/L for women or reproductive age and children under five years of age, respectively.

^d^Microcytic anaemia was classified for mothers and infants with anaemia (low haemoglobin) and low MCV (<81µm^3^ in trimester 1 and 3, < 82 µm^3^ in trimester 2; < 81 µm^3^ of pregnancy, < 79 µm^3^ for non-pregnant women, < 77 µm^3^ in infants at 1 year).

**Fig 3:**
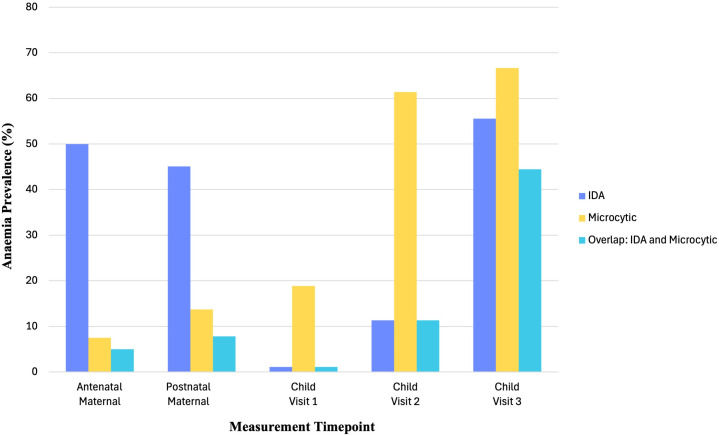
Relative Prevalence of Iron Deficiency Anaemia, Microcytic Anaemia, and Their Overlap in Mothers and Infants Across Study Visits.

In comparison to actual IDA classification estimates based on iron metrics (Table 6), microcytic anaemia was found to underestimate IDA in mothers during and after pregnancy (7.5% microcytic anaemia versus 50% IDA in pregnancy; 13.73% microcytic anaemia versus 45.10% IDA postnatally) and overestimate IDA in infants at 3–6 (18.89% microcytic anaemia versus 1.11% IDA) and 6–12 months (61.36% microcytic anaemia versus 11.36% IDA). Furthermore, there was poor overlap between IDA and microcytic anaemia classifications in mothers antenatally and postnatally, and in the first year of infancy (Fig 3). Of the mothers with microcytic anaemia during and after pregnancy, only 66.67% (2/3) and 57.14% (4/7) had IDA, respectively. Similarly, of the infants at 3–6 months and 6–12 months with microcytic anaemia, only 5.88% (1/17) and 18.52% (5/27) had IDA, respectively. However, the estimated prevalence of IDA and microcytic anaemia was similar in infants at 12–18 months (66.67% microcytic anaemia versus 55.56% IDA) and there was more overlap with 66.67% (8/12) of infants with microcytic anaemia also having IDA. This suggests that microcytic anaemia may be a better proxy for iron deficiency at this age.

## Discussion

This article is one of the first to characterise the prevalence and profile of anaemia, iron deficiency, and IDA with adjustment for inflammation in mothers (during and after pregnancy) and their infants in South Africa. The key findings highlight anaemia as a prevalent health concern, with the majority of cases being attributable to iron deficiency in mothers and infants by 12–18 months of age. Comparing WHO [27] and BRINDA [28] inflammatory adjustment methods revealed the extent to which iron deficiency and IDA may be underestimated in countries with a high burden of infection. Additionally, microcytic anaemia classification using MCV emerged as an unreliable proxy of IDA in mothers antenatally and postnatally, as well as infants under 1 year of age with inflammation.

### Maternal IDA

In this birth cohort, the prevalence of anaemia in pregnancy was found to be 34.74% with most mothers having mild or moderate anaemia. This is similar to previous estimates for LMICs [1], including South Africa, based on current census data [8], systematic reviews [9,10], and recent research from high-risk birth cohorts [12,13]. In turn, mothers with anaemia in pregnancy had a higher risk of having anaemia after childbirth, compared to those who were not anaemic in pregnancy. While the prevalence of postnatal maternal anaemia was still high (22.50%), it was lower than antenatal maternal anaemia; this was expected given that pregnancy represents a period of heightened metabolic demand [20,57,58]. Together, these findings corroborate the burden of anaemia in this context and confirm the WHO report of stagnated progress in reducing its prevalence [2,19].

While reported food insecurity was high with exacerbated effects due to COVID-19, anaemic and non-anaemic mothers (in pregnancy and postnatally) had similar access to food resources and comparable infant anthropometry. This suggests that anaemia in this sample was more likely to be associated with a micronutrient deficiency such as iron rather than gross malnutrition, reflecting “hidden hunger” [59,60]. This growing global phenomenon is most prominent in LMICs where food inequities foster a reliance on low-cost high-energy staples that lack nutritional value and dietary diversity [59]. While smoking and alcohol exposure was low, a higher proportion of anaemic mothers were found to be living with HIV. This is most likely due to the complex interplay between anaemia, iron metabolism, and viral pathogenesis [61]. Potential mechanisms for decreased erythropoiesis include bone marrow suppression as a direct viral effect of HIV, side-effects of antiretroviral medications such as Zidovudine, and a complication of opportunistic infections such as cytomegalovirus (CMV) in immunocompromised people [61,62]. While anaemia aetiology in HIV is multifaceted, the overarching mechanism is explained by chronic inflammation contributing to disrupted iron homeostasis (decreased absorption and increased sequestration) and, in turn, a functional iron deficiency characterised by limited iron bioavailability [22,23,61,63–65]. This association between maternal HIV and anaemia is consistent with other studies from South Africa [9–11], suggesting that inflammation may be an important aetiological factor for consideration.

In this sample, almost half (46.79%) of pregnant mothers had evidence of inflammation, mostly acute (raised *hs*CRP). It is possible that the lower prevalence of biomarkers for chronic inflammation (raised AGP) was attributable to immune modulation typical of the physiology of pregnancy [66,67]. The prevalence of postnatal maternal inflammation remained high (51.28%), with evidence for both acute (raised *hs*CRP) and chronic inflammation (raised AGP). As expected, AGP concentrations were positively correlated with serum ferritin levels in pregnancy, and both inflammatory biomarker concentrations were separately associated with serum ferritin levels in mothers postnatally. This is consistent with serum ferritin being a well-documented acute-phase reactant that becomes elevated in inflammatory states [24,25].

Adjustment for inflammation using Method A (WHO) and Method B (BRINDA) increased the estimated prevalence of antenatal maternal iron deficiency from 18.35% to 55.04% (36.69% increase) and 38.53% (20.18% increase), respectively. The estimated prevalence of maternal iron deficiency was lower postnatally than antenatally, but also increased from 11.97% to 46.58% (34.61% increase) and 25.21% (13.24% increase) when adjusting for inflammation using Methods A and B, respectively. Taken together, these findings emphasize the importance of adjusting serum ferritin for inflammation during and after pregnancy to avoid the underestimation of antenatal and postnatal maternal iron deficiency. Chronic inflammation (raised AGP) was most prevalent in mothers after childbirth, meaning that BRINDA adjustment for sTfR was necessary to avoid the overestimation of iron-deficient erythropoiesis in this period.

Using BRINDA-adjusted iron deficiency estimates, half of antenatal maternal anaemia cases were attributed to iron deficiency (IDA). In contrast, only 7.5% of antenatal maternal anaemia cases were classified as microcytic and, of these, only 66.67% were IDA. Similarly, while microcytic anaemia classification using traditional approaches suggested that only 13.73% of postnatal maternal anaemia cases were likely to be IDA, BRINDA-adjusted iron metrics demonstrated that 45.10% of anaemia cases were in fact attributable to iron deficiency. Furthermore, only 57.14% mothers with microcytic anaemia also had IDA postnatally. Overall, in the context of high inflammatory burden, using MCV as a proxy for iron deficiency during and after pregnancy may be less valid than during other times of life.

In sum, the prevalence of maternal anaemia in this South African community was high, particularly during pregnancy, with most cases being associated with iron deficiency. This was evident despite routine public sector antenatal iron supplementation and the National Food Fortification Programme, reinforcing the need to re-evaluate current screening protocols in South African healthcare systems [11]. The importance of adjusting iron metrics for inflammation is emphasized for mothers living with HIV who have been found to be at increased risk of IDA in this study and others [9–11]. Despite the high burden of anaemia, iron deficiency, and IDA, very few mothers had an accurate understanding of their own anaemia status at enrolment, with only four mothers (3.74%) in the anaemic group reporting their own status accurately. The implications of this suggest the need for programmes to empower communities and healthcare workers with knowledge around about the importance of nutrition, supplementation, and management of conditions including iron deficiency, substance use, and HIV, particularly during pregnancy and early life [3,39].

### Child IDA

The prevalence of child anaemia in this cohort was also high with 46.82% and 48.10% of infants being classified as anaemic at least once by the second and third study visit at approximate ages of 6–12 months and 12–18 months, respectively. However, the prevalence of new diagnoses decreased from 51.28% to 20.20% over the three postnatal study visits. This is consistent with research from South Africa, Kenya, and Togo suggesting that the risk of anaemia is greatest within the first year of life, progressively decreasing thereafter [9,68,69]. This pattern may be due to a rapid decline in haemoglobin and a change in globin chains after birth when the hypoxic uterine environment is exchanged for oxygen-rich air exposure [3].

In this study, early risk factors included malnutrition, as indicated by underweight classification, and prenatal alcohol exposure. Similarly, infants born to anaemic mothers were more likely to be anaemic by 12–18 months of age. This corroborates research from across Africa identifying antenatal maternal anaemia as an important predictor of childhood anaemia [69–71]. While child exposure to maternal HIV in utero was high, very few infants in this cohort were HIV-positive due to good maternal access to effective ART in pregnancy [72]. However, there was still high rates of infant inflammation, with approximately 20–43% of infants having positive inflammatory biomarkers, particularly for chronic inflammation (AGP > 1g/L). While there was no consistent relationship between acute or chronic inflammation and iron deficiency within the first 3–6 months of life, significant positive associations between *hs*CRP, AGP, and serum ferritin concentrations emerged after 6 months of age.

Based on unadjusted serum ferritin, a low estimated prevalence of iron deficiency was observed within the first 3–6 and 6–12 months, and adjustment for inflammation only marginally increased this. Furthermore, very few anaemia cases were attributed to iron deficiency in this early period (1.11% and 11.36%), despite the high proportion of anaemic infants being classified as having microcytic anaemia (18.89% and 61.36%). This may be due to the protective effects of foetal iron loading in pregnancy, with maternal iron stores typically sustaining iron demands in infants for the first 6 months when they are unable to acquire and absorb much iron from their diet [20,57,58]. Additionally, exclusive breastfeeding may have protective effects within the first 6 months of infancy due to the high bioavailability of iron in breastmilk (despite low iron concentrations) [3]. Overall, the biological mechanisms for anaemia in early life in this cohort are unclear with unstable associations between inflammatory biomarkers and iron metrics. This is most likely due to the fact that metabolic and immune processes are immature in infancy [73], with transitions between food sources from immune-protective breastmilk [74,75] to solid foods typically occurring in parallel [76]. Early infancy is a period of great flux due to the increased risk of infections, high prevalence of environmental exposures, and maturing physiological processes. Therefore, relationships between factors may be harder to disentangle during this 3–12-month window. In turn, MCV may be a poor proxy for IDA at this age given that only 5.88% and 18.52% of infants with microcytic anaemia were classified with IDA at 3–6 and 6–12 months, respectively.

By 12–18 months, iron deficiency anaemia patterns in our child cohort stabilised, becoming more similar to their mothers. At this timepoint, iron deficiency was more prevalent than earlier in infancy. This is consistent with research suggesting that the risk of iron deficiency is greatest between 6 and 24 months when maternal iron sources are depleted and infant growth is exponential [3]. Adjustment for inflammation using Methods A [27] and B [28] increased the estimated prevalence of iron deficiency from 19.79% to 31.25% (11.46% increase) and 32.29% (12.5% increase), respectively. As seen in the mothers, BRINDA-adjusted iron deficiency estimates accounted for approximately 50% of anaemia cases. However, MCV seemed to be a more reliable proxy for iron deficiency in infants at 12–18 months than those younger than a year, with similar prevalence estimates for microcytic anaemia (66.67%) and IDA (55.56%), and more overlap (66.67%). Lastly, given the high prevalence of chronic inflammation (AGP), it was necessary to adjust sTfR to avoid overestimating iron deficient erythropoiesis at this age.

The complex interplay between overlapping risk factors for childhood anaemia in Africa [68–71,77–79] highlights the importance of inflammatory biomarkers to support an improved understanding of risk, and to inform strategies for intervention at an individual and population level. This is particularly relevant at 12–18 months when the prevalence of IDA is likely to be underestimated in infants with active inflammation. However, further research on inflammation and the role of immune regulation in IDA in infants may provide further insight into this evolving relationship, particularly in the first year of life.

### Considerations for interpreting IDA

**Fig 4:**
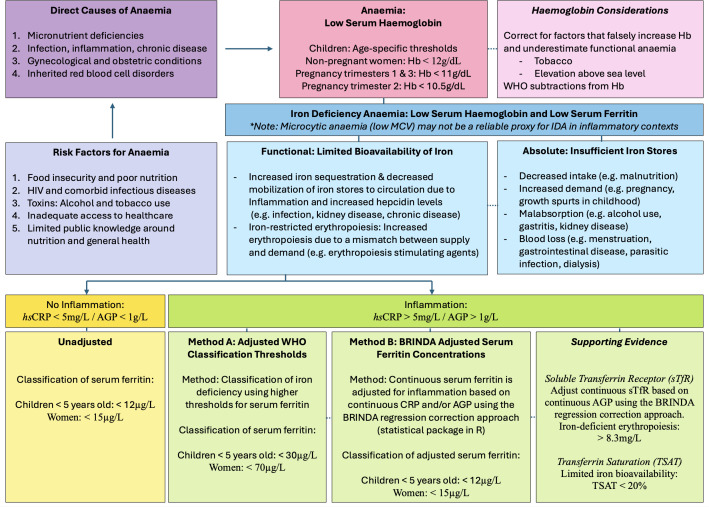
Considerations for Understanding and Interpreting Iron Deficiency Anaemia with Inflammation in Pregnancy and Infancy. Abbreviations: Hb, haemoglobin, IDA, iron Deficiency Anaemia, MCV, Mean Corpuscular Volume, highly sensitive C-Reactive Protein, AGP = Alpha-1-Acid Glycoprotein; sTfR, soluble transferrin receptor.

Based on this research as well as existing literature, we propose a conceptual framework (Fig 4) of important considerations for understanding and interpreting IDA metrics in the development of strategies for practice, with opportunities for future policy contributions. Fig 4 summarises key risk factors for anaemia including malnutrition [3,22,23], infectious diseases such as HIV [3,9,10,21,63], environmental exposures including alcohol and smoking [80,81], inadequate healthcare access [4], and limited public knowledge on nutrition [82,83]. Many of these drivers of anaemia [2] are more prevalent in LMICs due to high levels of poverty, poorer education, and more limited healthcare resources [1,6,7,37].

As previously described, anaemia and iron deficiency are classified using WHO thresholds for haemoglobin and serum ferritin, respectively, with age-specific and trimester-specific cutoffs for infants and women [27,46]. However, WHO guidelines include recommended subtractions to correct haemoglobin measurements for tobacco and altitude to avoid the underestimation of functional anaemia [46]. Following on from this, we recommend adjusting iron metrics for inflammation to account for functional iron deficiency due to decreased iron bioavailability in the context of infection. This is necessary as chronic inflammation may promote the expression of hepcidin, resulting in increased iron sequestration and decreased mobilisation of iron stores into circulation for use. Given that serum ferritin is an acute-phase reactant, it becomes transiently elevated in response to inflammation [24,25]. As demonstrated in this study, inflammatory biomarkers of acute (*hs*CRP > 5mg/L) or chronic inflammation (AGP > 1g/L) [50] are important for the valid interpretation of iron metrics when the burden of infectious disease is high [27,51].

Based on our results, we highlight two available methods for interpreting serum ferritin with adjustment for inflammation. Method A uses adjusted WHO thresholds to classify iron deficiency in cases with inflammation [27]. Method B uses the BRINDA regression approach to corrects serum ferritin based on *hs*CRP and AGP [28]. While Method A [27] is likely to be more sensitive, it is a less specific measure of iron deficiency due to its generalised approach. In comparison, Method B [28] is expected to have higher specificity as it corrects serum ferritin on an individual level, but the model is still being refined. As demonstrated in this research, additional supporting evidence may be derived from sTfR which serves as an indicator of iron-deficient erythropoiesis [29], but requires adjustment for inflammation using BRINDA [29]. While transferrin saturation (TSAT) was unavailable for inclusion in this study, it has also been identified as a reliable proxy for iron bioavailability [21–23,84].

## Conclusion and future directions

This study is one of the first to characterise IDA in South African mothers (during and after pregnancy) and infants with adjustment for inflammation. Overall, the results demonstrate the extent to which iron deficiency and IDA may be underestimated if inflammation is not accounted for when infectious burden is high. HIV was identified as a prevalent risk factor for anaemia in this study as seen across settings in South Africa [9–11], emphasizing the importance of adjustment for inflammation in this group. In contrast, our findings suggest that microcytic anaemia (based on MCV) may be a less useful proxy for IDA in mothers during and after pregnancy, as well as infants under 1 year of age with inflammation. Given the high prevalence of IDA and the even higher prevalence of iron deficiency, we highlight the value of adjusting serum ferritin using widely available inflammatory biomarkers (*hs*CRP and AGP). Furthermore, we emphasize the complex aetiology of anaemia in LMICs and propose informed considerations for understanding overlapping risk factors for the valid interpretation of relevant metrics in mothers and infants. These guidelines are likely to be key for improved screening, context-specific prevention efforts, and optimised interventions strategies around iron supplementation and management of infectious disease such as HIV [3,39].

While correction methods for inflammation adjustment are useful tools for detecting iron deficiency in countries with a high burden of infection, they are still based on existing reference ranges which may not be appropriate. The refinement of population-specific reference ranges is particularly necessary in communities of African descent, where it is estimated that genetic differences may translate into naturally lower haemoglobin concentrations [3]. Therefore, ongoing efforts to reduce IDA should include epidemiological data from LMICS (including pregnancy data) for representation across contexts with consideration of inflammation as a key aetiological contributor. Further studies should also include comprehensive data around iron supplementation adherence in pregnancy and parity to determine how current standard healthcare practices around screening and prevention can be improved, particularly in mothers who have had multiple consecutive births and may be at risk of iron depletion [11]. We recommend that this be conducted across settings in South Africa for more generalisable conclusions about the prevalence and profile of IDA, as well as the diagnostic efficacy of MCV in South Africa. Lastly, more research is required to understand the complex and evolving role of the immune system in IDA during early life.

Future work, such as the Gates Foundation funded ReMAPP (Redefining Maternal Anemia in Pregnancy and Postpartum) study, focused on identifying specific thresholds for anaemia and iron deficiency in pregnancy and infancy at a larger scale across LMICS, will be important in establishing appropriate thresholds for these periods [85]. In turn, this work may inform current intervention research including the Randomised controlled trial of the Effect of Intra-Venous Iron on Anaemia in Malawian Pregnant women (REVAMP; Gates Foundation) [86]. This multifactorial approach to understanding, preventing, and treating anaemia is important for ongoing efforts aimed at improving neurodevelopmental outcomes, particularly in LMICs such as South Africa where many infants are not reaching their full potential [14].

## Supporting information

S1 Text **Table A.** Classification of Child Anaemia by Age Across Study Visits. **Table B.** Inflammatory Biomarker Concentrations for Mothers and Infants with Serum Ferritin Across Study Visits. **Table C.** Maternal and Infant Sample Characteristics According to BRINDA-Adjusted Antenatal Maternal Iron Deficiency Status. **Table D.** Maternal and Infant Sample Characteristics According to BRINDA-Adjusted Postnatal Maternal Iron Deficiency Status at Study Visit 1 (±3–6 Months after Childbirth). **Table E.** Maternal and Infant Sample Characteristics According to BRINDA-Adjusted Child Iron Deficiency Status In Infants with Serum Ferritin at Study Visits 2 and 3.
(PDF)
