# Supplementary material for: Iron deficiency anaemia in mothers and infants with high inflammatory burden: Prevalence and profile in a South African birth cohort
Source: PLOS Glob Public Health. 2025 Jul 9;5(7):e0004174. doi: 10.1371/journal.pgph.0004174 (PMC12240388; doi:10.1371/journal.pgph.0004174)
Supplement: S1 Text — Table A. Classification of Child Anaemia by Age Across Study Visits. Table B. Inflammatory Biomarker Concentrations for Mothers and Infants with Serum Ferritin Across Study Visits. Table C. Maternal and Infant Sample Characteristics According to BRINDA-Adjusted Antenatal Maternal Iron Deficiency Status. Table D. Maternal and Infant Sample Characteristics According to BRINDA-Adjusted Postnatal Maternal Iron Deficiency Status at Study Visit 1 (±3–6 Months after Childbirth). Table E. Maternal and Infant Sample Characteristics According to BRINDA-Adjusted Child Iron Deficiency Status In Infants with Serum Ferritin at Study Visits 2 and 3. (PDF) [file pgph.0004174.s001.pdf]

SUPPORTING INFORMATION

IRON DEFICIENCY ANAEMIA IN MOTHERS AND INFANTS WITH HIGH INFLAMMATORY BURDEN: PREVALENCE AND PROFILE IN A SOUTH AFRICAN BIRTH COHORT

This PDF file includes:

Table A. Classification of Child Anaemia by Age Across Study Visits

Table B. Inflammatory Biomarker Concentrations for Mothers and Infants with Serum Ferritin Across Study Visits

Table C. Maternal and Infant Sample Characteristics According to BRINDA-Adjusted Antenatal Maternal Iron Deficiency Status

Table D. Maternal and Infant Sample Characteristics According to BRINDA-Adjusted Postnatal Maternal Iron Deficiency Status at Study Visit 1 (±3-6 Months after Childbirth)

Table E. Maternal and Infant Sample Characteristics According to BRINDA-Adjusted Child Iron Deficiency Status In Infants with Serum Ferritin at Study Visits 2 and 3

Table A. Classification of Child Anaemia by Age Across Study Visits

Child Age at Haemoglobin Measurement	Haemoglobin Concentration Threshold for Anaemia [a]	Number (%) of Haemoglobin Measures			Number (%) of Anaemic Observations		
		Study Visit 1 (*n* = 195)	Study Visit 2 (*n* = 173)	Study Visit 3 (*n* = 99)	Study Visit 1 (*n* = 100)	Study Visit 2 (*n* = 47)	Study Visit 3 (*n* = 20)
**0 – 3 days**	**<14g/dL**				0		
**3 days – 1 month**	**<15g/dL**				0		
**1 – 2 months**	**<11.5g/dL**				0		
**2 - 3 months**	**<9.4g/dL**	31 (15.90)			4 (4.00)		
**3 months – 6 months**	**<11.1g/dL**	164 (84.10)	9 (5.20)		96 (96.00)	5 (10.64)	
**6 months to 23 months**	**<10.5g/dL**		164 (94.80)	99 (100)	0	42 (89.36)	20 (100.00)
**24 – 59 months**	**<11g/dl**				0	0	
**60 months +**	**<11.5**				0	0	

[a] Reference ranges for children between 0 and 6 months were obtained from GSH/UCT Pathology Laboratory guidelines, Groote Schuur Hospital, National Health Laboratory Service (Western Cape); effective date 23 January 2003. WHO guidelines were used for children over 6 months.
SI conversion factor: To convert to haemoglobin grams per litre, multiply by 10.

Table B. Inflammatory Biomarker Concentrations for Mothers and Infants with Serum Ferritin Across Study Visits

Inflammatory Biomarker	Measurement [a]				
	Antenatal Maternal (*n* = 109)	Postnatal Maternal (*n* = 234)	Child Visit 1 (*n* = 196)	Child Visit 2 (*n* = 158)	Child Visit 3 (*n* = 96)
***hs*CRP (mg/L)** [b]	8.20 (10.28) [0.3 – 65.3]	4.87 (7.02) [0.10 – 58.71]	2.54 (6.92) [0.10 – 51.34]	3.5 (9.41) [0.10 – 64.40]	3.75 (9.42) [0.10 – 56.05]
**AGP (g/L)** [b]	0.55 (0.23) [0.27 – 1.78]	0.97 (0.34) [0.29 – 2.13]	0.74 (0.32) [0.29 – 1.99]	0.91 (0.34) [0.34 – 2.23]	1.03 (0.55) [0.24 – 3.81]

Abbreviations. *hs*CRP = highly sensitive C-Reactive Protein, AGP = Alpha-1Acid Glycoprotein; sTfR, soluble transferrin receptor
[a] Values for continuous variables are presented as: mean (standard deviation) [range].
[b] Missing Values: Antenatal maternal *hs*CRP (*n*=4), postnatal maternal *hs*CRP (*n* = 8), infant *hs*CRP (*n* = 6 at study visit 1, *n* = 5 at study visit 3), infant AGP (*n* = 3 at study visit 1, *n* = 2 at study visit 2, *n* = 3 at study visit 3).

Table C. Maternal and Infant Sample Characteristics According to BRINDA-Adjusted Antenatal Maternal Iron Deficiency Status

Variable[a]	Total sample (*n* = 109)		
	Maternal Iron Deficiency [b] (*n* = 42)	No Maternal Iron Deficiency [b] (*n* = 67)	*p*
**Maternal Characteristics**			
Adjusted serum ferritin (μg/L) [b,e]	11.09 (2.51) [5.47 – 14.81]	34.86 (23.61) [15.03 – 139.28]	<0.001***
Adjusted sTfR (mg/L) [c,e]	5.56 (2.3) [2.30 – 12.39]	4.39 (1.34) [1.73 – 8.81]	0.004**
*hs*CRP (mg/L)	6.63 (7.83) [0.30 – 41.30]	9.21 (11.52) [0.40 – 65.30]	0.211
AGP (g/L)	0.50 (0.17) [0.29 – 0.91]	0.59 (0.26) [0.27 – 1.78]	0.064
Monthly household income (ZAR) [f,g]			
<1000	8 (19.04)	14 (20.90)	0.979
1000-5000	18 (42.86)	29 (43.28)	
5000-10000	6 (14.29)	13 (19.40)	
>10000	1 (2.38)	1 (1.49)	
Education [f]			
Primary	0 (0.00)	2 (2.99)	0.517
Some secondary	18 (42.86)	30 (44.78)	
Completed secondary	21 (50.00)	27 (40.30)	
Some tertiary	1 (2.38)	6 (8.96)	
Completed tertiary	2 (4.76)	2 (2.99)	
Employed	15 (35.71)	21 (31.34)	0.637
Age at enrolment (years)	28.10 (5.53) [19.6 – 40.3]	29.16 (5.88) [19.0 – 40.4]	0.351
Food insecurity	22 (52.38)	38 (56.72)	0.658
COVID-19 effect on food insecurity [g]			
No disruption	15 (35.71)	25 (37.31)	0.526
Some disruption	7 (16.67)	11 (16.42)	
Extreme disruption	10 (23.81)	9 (13.43)	
Smoking during pregnancy [f]	0 (0.00)	1 (1.49)	1
Alcohol during pregnancy [f]	0 (0.00)	3 (4.48)	0.283
Depression during pregnancy	12 (28.57)	11 (16.42)	0.130
HIV infection during pregnancy [g]	19 (45.24)	25 (37.31)	0.387
**Infant Characteristics at Birth**			
Sex (male) [g]	23 (54.76)	23 (34.33)	0.027*
Gestational age at birth (weeks) [g]	39.63 (1.12) [37 - 42]	38.95 (1.85) [33 - 41]	0.092
HIV infection [g]	0 (0.00)	0 (0.00)	n/a
Birth weight (g) [d,g]	3283.33 (416.24) [2400 - 4360]	3160.89 (522.82) [1780 – 4120]	0.240
Birth length (cm) [d,g]	50.47 (2.80) [44 - 57]	50.02 (3.35) [41 – 57]	0.503
Birth head circumference (cm) [d,g]	35.07 (1.36) [32 – 38]	34.46 (1.69) [30.5 – 37.5]	0.075

Abbreviations. ZAR, South African Rand; HIV, Human Immunodeficiency Virus; COVID-19, Coronavirus Disease 2019; g, grams; cm, centimetres; *hs*CRP = highly sensitive C-Reactive Protein, AGP = Alpha-1Acid Glycoprotein; sTfR, soluble transferrin receptor.

[a] Values for continuous variables are presented as: mean (standard deviation) [range]. Values for categorical variables are presented as: count (%).

[b] Serum ferritin concentrations were adjusted for inflammation using the BRINDA regression correction approach. Antenatal maternal iron deficiency classified as adjusted serum ferritin concentrations <15µg/L

[c] sTfR concentrations were adjusted for inflammation using the BRINDA regression correction approach.

[d] The birth anthropometric measurements were conducted by trained labour staff in the ward. Infant length and head circumference were measured in cm to the nearest completed 0.5cm and weight was measured in kgs.

[e] Levene's test was significant. *T*-test results were interpreted based on equal variance not assumed.

[f] Fisher's exact test result interpreted due to one or more cells having an expected count of less than 5.

[g] Missing values: household income (n = 19), effect of COVID-19 on food insecurity (n = 32), maternal HIV (n = 2), infant sex (n = 16), infant gestational age (n = 39), infant HIV (n = 75), infant birth weight (n = 17), infant birth length (n = 18), infant birth head circumference (n = 18).

*p is significant at <0.05, ** p is significant at <0.01, ***p is significant at <0.001.

Table D. Maternal and Infant Sample Characteristics According to BRINDA-Adjusted Postnatal Maternal Iron Deficiency Status at Study Visit 1 (±3-6 Months after Childbirth)

Variable[a]	Total sample (*n* = 234)		
	Maternal Iron Deficiency [b] (*n* = 59)	No Maternal Iron Deficiency [b] (*n* = 175)	*p*
**Maternal Characteristics**			
Adjusted serum ferritin (μg/L) [b,e]	9.55 (3.11) [3.14 – 14.98]	35.89 (20.28) [15.12 – 115.53]	<0.001***
Adjusted sTfR (mg/L) [c]	4.58 (1.45) [2.23 – 12.01]	3.89 (1.11) [1.78 – 7.92]	<0.001***
*hs*CRP (mg/L) [g]	4.37 (5.49) [0.10 – 30.56]	5.03 (7.46) [0.10 – 58.71]	0.539
AGP (g/L)	0.94 (0.33) [0.29 – 1.87]	0.97 (0.34) [0.38 – 2.13]	0.538
Monthly household income (ZAR) [f,g]			
<1000	10 (16.95)	33 (18.86)	0.476
1000-5000	27 (45.76)	76 (43.43)	
5000-10000	17 (28.81)	39 (22.29)	
>10000	1 (1.69)	11 (6.29)	
Education [f]			
Primary	2 (3.39)	4 (2.29)	0.575
Some secondary	30 (50.85)	78 (44.57)	
Completed secondary	18 (30.51)	72 (41.14)	
Some tertiary	5 (8.47)	13 (7.43)	
Completed tertiary	4 (6.78)	8 (4.57)	
Employed [g]	15 (25.42)	65 (37.14)	0.090
Age at study visit 1 (years) [g]	28.61 (5.85) [18 – 42]	29.05 (5.50) [18 – 44]	0.601
Food insecurity	37 (62.71)	91 (52)	0.153
COVID-19 effect on food insecurity [g]			
No disruption	33 (55.93)	97 (55.43)	0.957
Some disruption	12 (20.34)	38 (21.71)	
Extreme disruption	11 (18.64)	36 (20.57)	
Smoking at enrolment [f]	2 (3.39)	7 (4.00)	1
Alcohol at enrolment [f]	1 (1.69)	12 (6.86)	0.193
Depression at enrolment	9 (15.25)	31 (17.71)	0.664
HIV infection at enrolment	23 (38.98)	58 (33.14)	0.415
**Infant Characteristics at Study Visit 1**			
Age (months) [g]	3.83 (0.81) [2.14 – 5.23]	3.77 (0.78) [2.01 – 5.85]	0.631
Sex (male)	21 (35.59)	100 (57.14)	0.004**
HIV infection [g]	0 (0.00)	0 (0.00)	n/a
Microcephaly [d,f,g]	0 (0.00)	1 (0.57)	1
Underweight [d,f,g]	2 (3.39)	5 (2.86)	1
Stunting [d,g]	7 (11.86)	23 (13.14)	0.798

Abbreviations. ZAR, South African Rand; HIV, Human Immunodeficiency Virus; COVID-19, Coronavirus Disease 2019; g, grams; cm, centimetres; . *hs*CRP = highly sensitive C-Reactive Protein, AGP = Alpha-1Acid Glycoprotein; sTfR, soluble transferrin receptor.

[a]Values for continuous variables are presented as: mean (standard deviation) [range]. Values for categorical variables are presented as: count (%).

[b] Serum ferritin concentrations were adjusted for inflammation using the BRINDA regression correction approach. Iron deficiency classified as adjusted serum ferritin concentrations <15μg/L

[c] sTfR concentrations were adjusted for inflammation using the BRINDA regression correction approach.

[d] The anthropometric measurements were conducted by trained research staff. Child weight and length measurements were converted to z-scores based on age and sex using Anthro software for WAZ, HAZ, and HCZ. Infants were classified as underweight, stunted, or having microcephaly if they had z-scores of less than -2 SDs.

[e] Levene's test was significant. *T*-test results were interpreted based on equal variance not assumed.

[f] Fisher's exact test result interpreted due to one or more cells having an expected count of less than 5.

[g] Missing values: maternal *hs*CRP at study visit 1 ($n = 8$), maternal age at study visit 1 ($n = 2$), maternal employment ($n = 2$), household income ($n = 20$), effect of COVID-19 on food insecurity ($n = 7$), infant age at study visit 1 ($n = 52$), infant HIV ($n = 162$), infant microcephaly at study visit 1 ($n = 5$), infant underweight at study visit 1 ($n = 5$), infant stunting at study visit 1 ($n = 8$).

**p* is significant at <0.05, ** *p* is significant at <0.01, ***p* is significant at <0.001.

Table E. Maternal and Infant Sample Characteristics According to BRINDA-Adjusted Child Iron Deficiency Status In Infants with Serum Ferritin at Study Visits 2 and 3

Variable [a]	Study Visit 2 (±6-12 months; n = 158)			Study Visit 3 (±12-18 months; n = 96) [g]		
	Child Iron Deficiency [b] (n = 10)	No Child Iron Deficiency [b] (n = 148)	p	Child Iron Deficiency [b] (n = 31)	No Child Iron Deficiency [b] (n = 64)	p
**Infant Characteristics**						
Adjusted Serum Ferritin (μg/L) [b,e,g]	9.44 (1.85) [6.44 – 11.68]	38.10 (25.64) [12.10 – 200.71]	<0.001***	8.15 (2.66) [2.55 – 11.88]	29.38 (18.35) [12.32 – 107.59]	<0.001***
Adjusted sTfR (mg/L) [c,e,g]	10.31 (3.29) [7.10 – 17.43]	6.80 (1.46) [4.17 – 12.23]	0.013*	7.13 (3.54) [1.84 – 21.12]	5.69 (1.45) [3.56 – 10.02]	0.042*
*hs*CRP (mg/L) [g]	2.19 (2.80) [0.10 – 7.54]	3.59 (9.70) [0.10 – 64.4]	0.651	3.20 (10.11) [0.10 – 56.05]	4.03 (9.12) [0.10 – 52.83]	0.692
AGP (g/L) [e]	0.99 (0.38) [0.51 – 1.56]	0.91 (0.34) [0.34 – 2.23]	0.513	0.96 (0.65) [0.24 – 3.81]	1.06 (0.50) [0.34 – 2.85]	0.435
Age (months) [e,g]	8.59 (1.30) [6.15 – 10.55]	8.55 (1.60) [5.29 – 12.20]	0.938	13.99 (1.02) [12.43 – 15.88]	14.23 (1.36) [12.0 – 18.05]	0.386
Sex (male) [f,g]	7 (70.00)	73 (49.32)	0.328	21 (67.74)	35 (54.69)	0.225
HIV infection [f,g]	0 (0.00)	1 (0.68)	1	1 (3.23)	0 (0.00)	0.341
Microcephaly [d,f,g]	0 (0.00)	4 (2.70)	1	0 (0.00)	1 (1.56)	1
Underweight [d,f,g]	0 (0.00)	5 (3.38)	1	1 (3.23)	1 (1.56)	0.548
Stunting [d,f,g]	0 (0.00)	16 (10.81)	0.600	2 (6.45)	9 (14.06)	0.495
**Maternal Characteristics**						
Monthly household income (ZAR) [f,g]						
<1000	2 (20.00)	24 (16.22)	0.209	8 (25.81)	13 (20.31)	0.928
1000-5000	6 (60.00)	70 (47.30)		15 (48.39)	33 (51.56)	
5000-10000	0 (0.00)	36 (24.32)		6 (19.35)	10 (15.63)	
>10000	1 (10.00)	8 (5.41)		1 (3.23)	3 (4.69)	
Education [f]						
Primary	0 (0.00)	3 (2.03)	0.806	1 (3.23)	2 (3.13)	0.208
Some secondary	6 (60.00)	66 (44.59)		18 (58.06)	27 (42.19)	
Completed secondary	4 (40.00)	57 (38.51)		8 (25.81)	27 (42.19)	
Some tertiary	0 (0.00)	16 (10.81)		4 (12.90)	4 (6.25)	
Completed tertiary	0 (0.00)	6 (4.10)		0 (0)	4 (6.25)	
Employed [f,g]	2 (20.00)	48 (32.43)	0.720	10 (32.26)	24 (37.50)	0.695
Age at enrolment (years) [g]	29.26 (5.64) [19.7 – 36.2]	29.23 (5.69) [18.1 – 42.6]	0.988	30.10 (5.83) [18.9 – 38.5]	28.67 (5.90) [18.0 – 44.1]	0.269
Food insecurity [f,g]	4 (40.00)	84 (56.76)	0.340	12 (38.71)	41 (64.06)	0.020*
COVID-19 effect on food insecurity [f,g]						
No disruption	3 (30.00)	64 (43.24)	0.217	10 (32.26)	27 (42.19)	0.022*
Some disruption	1 (10.00)	23 (15.54)		2 (6.45)	13 (20.31)	
Extreme disruption	4 (40.00)	24 (16.22)		11 (35.48)	9 (14.10)	
Smoking at enrolment [f,g]	0 (0.00)	6 (4.05)	1	1 (3.23)	6 (9.38)	0.421

Alcohol at enrolment [f,g]	1 (10.00)	9 (6.08)	0.490	2 (6.45)	7 (10.94)	0.713
Depression at enrolment [f,g]	3 (30.00)	26 (17.57)	0.393	6 (19.35)	10 (15.63)	0.649
HIV infection at enrolment [f,g]	3 (30.00)	57 (38.51)	0.743	15 (48.39)	28 (43.75)	0.670

Abbreviations. ZAR, South African Rand; HIV, Human Immunodeficiency Virus; COVID-19, Coronavirus Disease 2019; g, grams; cm, centimetres; . *hs*CRP = highly sensitive C-Reactive Protein, AGP = Alpha-1Acid Glycoprotein; sTfR, soluble transferrin receptor.

[a]Values for continuous variables are presented as: mean (standard deviation) [range]. Values for categorical variables are presented as: count (%).

[b] Serum ferritin concentrations were adjusted for inflammation using the BRINDA regression correction approach. Iron deficiency classified as adjusted serum ferritin concentrations <12µg/L.

[c] sTfR concentrations were adjusted for inflammation using the BRINDA regression correction approach.

[d] The anthropometric measurements were conducted by trained research staff. Child weight and length measurements were converted to z-scores based on age and sex using Anthro software for WAZ, HAZ, and HCZ. Infants were classified as underweight, stunted, or having microcephaly if they had z-scores of less than -2 SDs..

[e]Levene's test was significant. T-test results were interpreted based on equal variance not assumed.

[f] Fisher's exact test result interpreted due to one or more cells having an expected count of less than 5.

[g] Missing values: BRINDA adjusted serum ferritin (n = 1 at study visit 3), infant *hs*CRP (n = 5 at study visit 3), infant AGP (n = 2 at study visit 2, n = 3 at study visit 3), infant adjusted sTfR (n = 3 at study visit 2, n = 4 at study visit 3), infant age (n = 7 at study visit 2, n = 3 at study visit 3), infant sex (n = 1 at study visit 3), infant HIV (n = 104 at study visit 2, n = 55 at study visit 3), infant microcephaly (n = 1 at study visit 3), infant underweight (n = 1 at study visit 3), infant stunting (n = 2 at study visit 2, n = 1 at study visit 3), household income (n = 11 at study visit 2, n = 7 at study visit 3), maternal employment (n = 1 at study visit 2, n = 2 and study visit 3), maternal education (n = 1 at study visit 3), maternal age at enrolment (n = 1 at study visit 3), food insecurity (n = 1 at study visit 3), effect of COVID-19 on food insecurity (n = 39 at study visit 2, n = 24 at study visit 3), maternal PTE (n = 1 at study visit 3), maternal PAE (n = 1 at study visit 3), maternal depression at enrolment (n = 1 at study visit 3), maternal HIV (n = 1 at study visit 3).

*p is significant at <0.05, ** p is significant at <0.01, ***p is significant at <0.001.
